# Assessing the Potential of Multispectral UAV-Derived Vegetation Indices for Estimating Water Use of Taro (*Colocasia esculenta*) Under Different Weed Management Practices

**DOI:** 10.3390/plants15182857

**Published:** 2026-09-18

**Authors:** Knowledge Muchaonyerwa, Maqsooda Mahomed, Shaeden Gokool, Alistair Clulow, Gary Denton, Kyle Reddy, Richard Kunz

**Affiliations:** 1 Centre for Water Resources Research, School of Agriculture and Science, University of KwaZulu-Natal, Pietermaritzburg 3209, South Africa; mahomedm@ukzn.ac.za (M.M.); gokools@ukzn.ac.za (S.G.); clulowa@ukzn.ac.za (A.C.); reddykyle406@gmail.com (K.R.); kunzr@ukzn.ac.za (R.K.); 2 South African Environmental Observation Network (SAEON), Grasslands-Forests-Wetlands Node, Pietermaritzburg 3201, South Africa; 3 Discipline of Agrometeorology, School of Agriculture and Science, University of KwaZulu-Natal, Pietermaritzburg 3209, South Africa; garydenton44@gmail.com

**Keywords:** evapotranspiration, taro, unmanned aerial vehicles, Google Earth engine, weed management

## Abstract

Water availability is typically a limiting factor in rainfed sub-Saharan farming systems. Smallholder farmers in this region grow neglected and underutilized crops (NUCs) such as taro to supplement food shortages. Understanding the water use of NUCs could contribute to improving productivity. Traditional methods of monitoring actual evapotranspiration (ETa) are point-based and do not capture the variability in smallholder fields. In contrast, empirical models that use vegetation indices derived from multispectral unmanned aerial vehicles (UAVs) have demonstrated the potential to provide reliable spatial variability of ETa. This study evaluated the viability of a VI-based empirical model to estimate the ETa of taro under different weed management practices in a smallholder farm using multispectral UAV imagery. Three treatments were tested: a consistently weeded plot, a plot that remained unweeded throughout the season, and an intermediate field that was partially managed for a short period after planting. A validated VI-based model that used the enhanced vegetation index (EVI2) as a proxy for the crop coefficient (Kc) was combined with reference evapotranspiration (ETo) to determine ETa on the three taro plots. The results demonstrated that the weeded plot used 27.0% less water, while the unweeded field used 35.5% more water than the intermediate field (representing the farmer’s typical integrated weed management practices throughout the growing season). Although ETa in the unweeded plot represents combined evapotranspiration from taro, weeds, and the soil surface rather than taro water use alone, the findings support an association between reduced crop–weed competition, lower evapotranspiration, and increased taro yield. The VI-based empirical models derived from multispectral UAV imagery showed great potential for promoting water use efficiency through integrated weed management (IWM) in smallholder farms.

## 1. Introduction

The growing population demands an increase in agricultural productivity to address potential food security concerns. Diversifying production through the promotion of neglected and underutilized crops (NUCs) such as taro (*Colocasia esculenta*) has been among the plausible responses [1,2,3]. Although the crop has been particularly under-researched, recent scientific evidence acknowledges its high nutritional value and resilience to climate variability [1,4]. According to Gerrano et al. [5], most taro producers in South Africa are smallholder farmers located in the Eastern Cape, KwaZulu-Natal, Mpumalanga, and Limpopo provinces. Taro serves as an affordable source of essential nutrients for resource-limited households. These farmers primarily depend on rainfall, making it critical to quantify the water use of taro to support improved management practices and maximize yields [6].

Much of South Africa falls within a semi-arid zone, receiving a mean annual precipitation (MAP) of approximately 465 mm [7], with climate projections pointing to heightened drought risk, especially in already dry regions [8,9,10]. Interventions to enhance water-use efficiency in agriculture through sustainable irrigation scheduling, improved monitoring systems, and the selection of climate-resilient genotypes have expanded as a response [11]. However, these efforts have largely concentrated on commercially dominant crops, leaving NUCs excluded from the research and investment needed to support diversification [12], thus necessitating pragmatic approaches that can be applied within smallholder communities [13].

Crop water use, commonly referred to as actual evapotranspiration (ETa), is calculated as a product of the crop coefficient (Kc) and reference evapotranspiration (ETo) [14,15,16]. The most reliable ways of obtaining ETa have been through in situ-based techniques such as eddy covariance, Bowen ratio methods, and surface renewal [17,18]. However, these point-based approaches are limited by high equipment costs, the need for specialized expertise, and their application over homogenous areas, which hinders their capability to account for spatial variability. Over the years, technological advancements have incorporated remote sensing platforms such as open-source satellite imagery (Landsat, Sentinel, and MODIS) in estimating ETa [16,19]. Although these datasets have been beneficial for multi-temporal scales, the overpass time, spatial resolution, cloud and dust interference in the atmosphere are some challenges that inhibit their suitability in smallholder farms. On the other hand, unmanned aerial vehicles (UAVs) have demonstrated great potential to contribute towards addressing these limitations [20].

There is currently no consensus on a gold-standard algorithm for estimating ETa in smallholder farms. However, the most common algorithms in remotely sensed ETa research are the surface energy balance system (SEBS), the surface energy balance algorithm for land (SEBAL), and mapping evapotranspiration at high resolution with internalized calibration (METRIC). They all rely on land surface temperature (LST) (captured by the thermal sensor) data to define wet and dry pixel ranges in a region of interest (ROI) [16,21,22]. Their thermal sensor and parameterization demand make them impractical for resource-constrained settings, motivating simpler alternatives with fewer input requirements.

To date, one of the simplest ways of obtaining ETa estimates is through vegetation index (VI)-based empirical models that rely on normalized difference vegetation index (NDVI) and the enhanced vegetation index (EVI2) as proxies for Kc. So far, these methods have been validated in satellite imagery studies [23,24,25]. There is limited evidence of these models being validated using UAV imagery, apart from Yacoob et al. [26] whose findings supported the EVI2 based model as the best performing in a small-scale sugarcane field. To date, there is no study that has evaluated VI-based ETa models using UAV imagery for NUCs, a gap that this study addresses for taro.

A second gap concerns weed management. Taro has a slow initial growth period in which it can take up to 49 days for the crop to emerge and 300 days to reach physiological maturity. This makes it a poor weed competitor for water, nutrients, light, and space [27]. Yet many UAV-based weed studies have primarily focused on yield loss, overlooking the impact of weeds on the water uptake. Therefore, how weed management affects taro’s water use, yields, and economic outcomes remain under-researched.

Addressing these gaps and utilizing cloud computing infrastructure such as Google Earth Engine contributes towards efficient processing of multispectral imagery [28]. This study evaluated the viability of a VI-based empirical model to estimate the ETa in plots where taro was grown under different weed management practices in a smallholder farm using multispectral UAV imagery. To fulfill the research aim, this study pursued three specific objectives: (1) to evaluate the performance of VI-based ETa empirical models using in situ data; (2) to determine the potential of an optimal VI-based empirical model for estimating taro water use under different management practices; and (3) to assess the impact of various weed management practices on taro yield and economic value.

## 2. Results

### 2.1. Identifying the Optimal VI-Based ETa Models Based on Statistical Metrics

VI-based estimates were validated against in situ data on the specific days which UAV images were captured. According to the ranking, the most optimal models were ETa-EVI2 (Kc), ETa-MEVI2, and ETa-NDVI, ETa-NDVI (scaled) in Site A, and ETa-MEVI2, ETa-EVI2 (scaled), ETa-NDVI, and ETa-EVI2 (Kc) in Site B ranked 1^st^, 2^nd^, 3^rd^, and 4^th^, respectively, as shown in Table 1. The weakest models were ETa-NDVI (Kc), ETa-EVI2, and ETa-NDVI (scaled), ranking 7, 6, and 4 in Site A, and 6, 5, and 7 in Site B. Site A had strong R^2^ values that ranged between 0.83 and 0.87 compared to Site B, which had 0.49 and 0.57, respectively. The error metrics highlighted poor model performance on both sites. Although optimal models were identified based on the statistical ranking, which was influenced by the weighting of the metrics. All models were carried forward for validation against the in situ daily data throughout the season for site A and B (Section 2.2).

### 2.2. The Validation of VI-Based Empirical Models with Eddy Covariance Data

**Figure 1:**
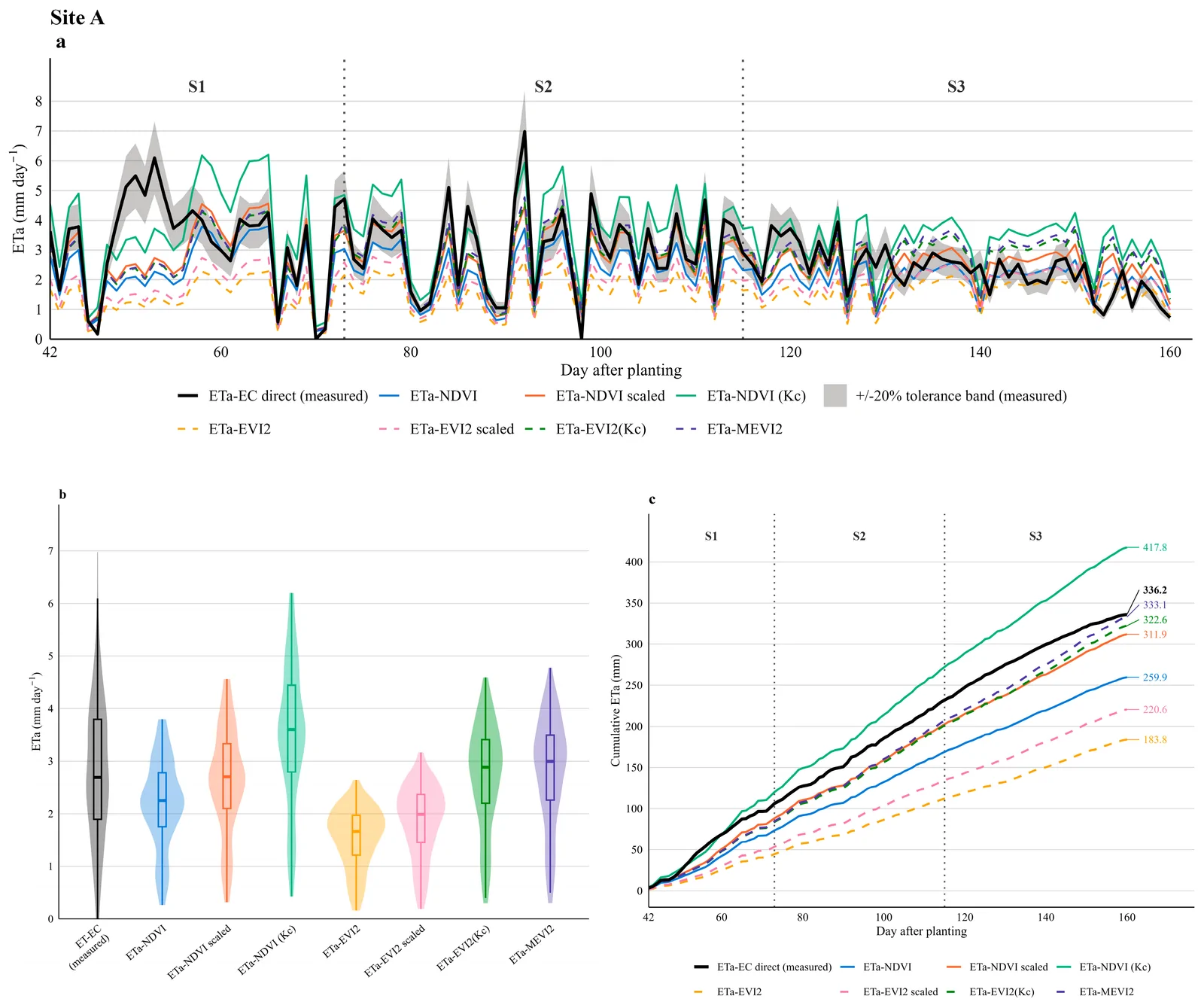
Graphs illustrating (**a**) temporal graph showing the relationship between daily measured ETa by the EC flux tower and estimates from the VI-based empirical models between day 42 and 160 after planting; (**b**) the violin plots of the daily distribution of measured and estimated ETa; (**c**) accumulated ETa derived from both observed EC flux tower data and VI-based model estimates, in Site A.

The in situ data that was used in Site A were collected between 1 January 2023 and 30 April 2023. Overall, the magnitude of variation in observed and simulated ETa was comparable Figure 1a. Three of the four optimal models identified in Section 2.1 showed close alignment with measured ETa. However, their ranking order differed. Based on total accumulated ETa throughout the season, ETa-MEVI2 ranked first, followed by ETa-EVI2 (Kc), and ETa-NDVI (scaled) (Figure 1b). The measured data during the observed period was 336.2 mm, while the models were 333.1 mm, 322.6 mm, and 311.9 mm, respectively, which was an underestimation of 0.9%, 4.0%, and 7.2%. Significant differences were noted between days 48 and 53. The range for measured ETa was 2.21–6.09 mm, while the ranges for the simulated were 2.23–2.60 mm (ETa- MEVI2, and ETa-EVI2 (Kc)), and 2.23–2.65 mm (ETa-NDVI (scaled), respectively, as depicted in Figure 1a. All three models overestimated towards the end of the season, with the ETa-NDVI (scaled) remaining the closest to the observed data (Figure 1a). The poorest performing models were ETa-NDVI (Kc), ETa-EVI2 (scaled), and ETa-EVI2, with a cumulative overestimation of 24.3% (417.8 mm), while the other two underestimated by 34.3% (220.6 mm), and 45.3% (183.8) (Figure 1c).

**Figure 2:**
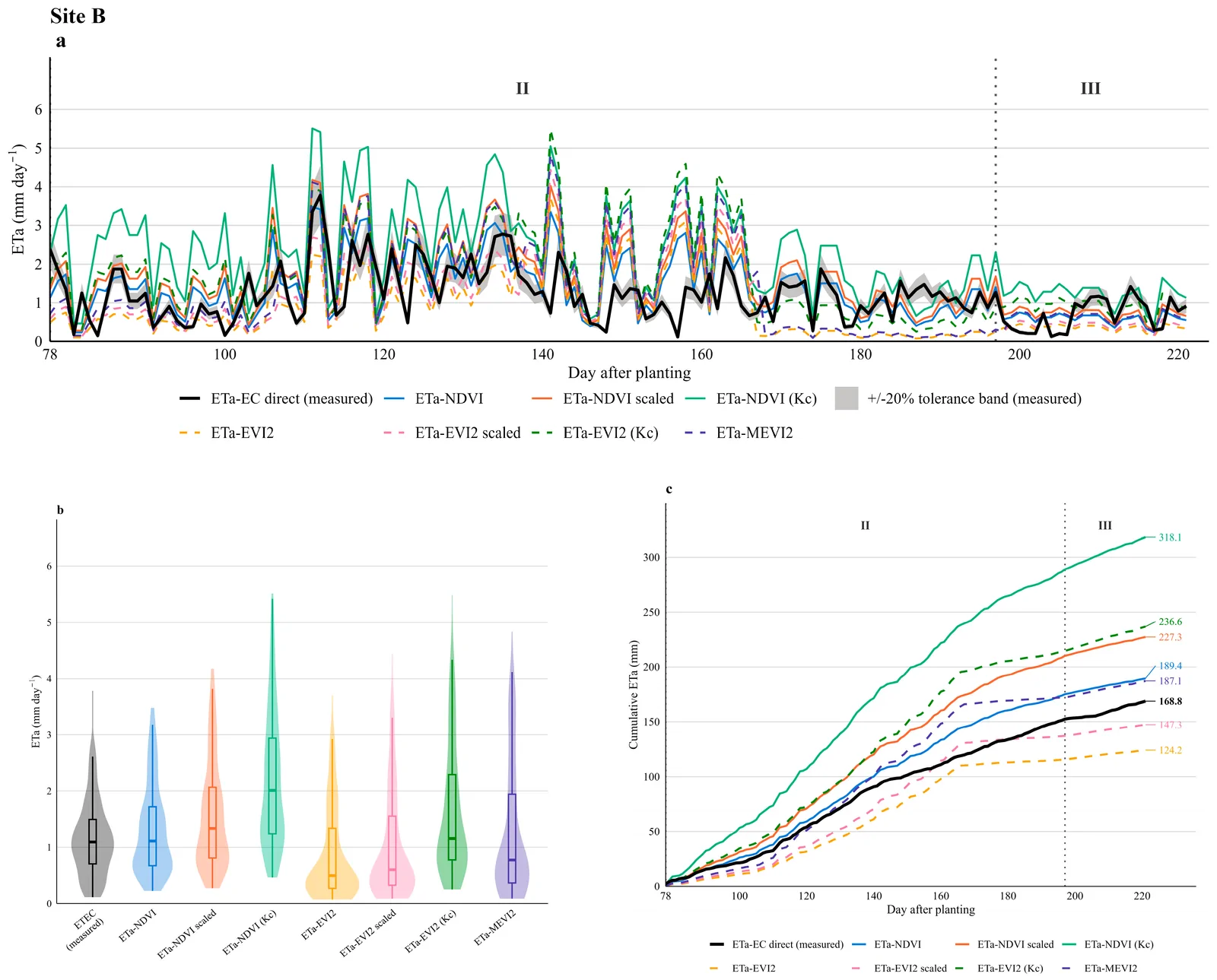
Graphs illustrating (**a**) temporal relationship between daily measured ETa from the eddy covariance flux tower and estimates from VI-based empirical models between days 78 and 221 after planting; (**b**) the violin plots of the daily distribution of measured and estimated ETa; (**c**) accumulated ETa derived from both observed EC flux tower data and VI-based model estimates, in Site B.

The in situ data used in Site B were collected between 1 February 2022 and 24 June 2022. The simulated outputs generally overestimated ETa for most of the recorded period except for ETa-EVI2 and ETa-EVI2 (scaled) (Figure 2a). The strongest candidates for the optimal models at this site were ETa-MEVI2, ETa-NDVI, and ETa-EVI2 (scaled): ETa-MEVI2 and ETa-NDVI recorded cumulative overestimations of 10.9% (187.1 mm) and 12.2% (189.4 mm), while ETa-EVI2 (scaled) underestimated by 12.7% (147.3 mm). The temporal dynamics for optimal models were broadly consistent with the eddy covariance (EC) observations with ETa-NDVI having the highest violin plot similarity (Figure 2b). Despite ETa-MEVI2 having the closest cumulative ETa to the in situ data (Figure 2c), it is important to note that ETa-NDVI had the best day-to-day tracking of the three models, with the highest percentage of days within the tolerance band (44.4%) compared to ETa-MEVI2 and ETa-EVI2 (scaled) that recorded 18.8% and 20.1%, respectively (Table 2).

There were three distinct growth phases (S1, S2, and S3) in Site A, while site B had two (II and III) that were recorded (Table 2 and Table 3). The empirical models were evaluated against EC-derived ETa values to assess their performance across the different stages (Table 2 and Table 3). The optimal models at Site A: ETa-MEVI2, ETa-EVI2 (Kc), and ETa-NDVI (scaled) underestimated by 20.5%, 21.2%, and 16.6%, respectively, during the early growth phase (S1, 42–72 DAP) with all three models recording 67.7% of days within the tolerance band. During the mid-growth stage (S2, 73–114 DAP), the underestimation narrowed to 3.4%, 7.3%, and −9.8% corresponded to 88.1%, 81.0%, and 78.6% of days that were within the tolerance band. The late growth stage (S3, 120–160 DAP) had a different story: the models overestimated by 20.6%, 16.1%, and 4.7%, respectively, with 41.3%, 47.8%, and 60.9% of days within the tolerance band (Table 2).

At Site B, the performance of the models showed contrasting behavior across Phase II (78–196 DAP) and III (197–221 DAP). In Phase II, two of the optimal models ETa-MEVI2, and ETa-NDVI, overestimated by 13.8% and 14.9%, while ETa-EVI2 (scaled) underestimated by 9.5%; the distribution of days within the tolerance band were 16.8%, 50.4%, and 22.7% for ETa-MEVI2, ETa-NDVI, and ETa-EVI2 (scaled). In Phase III, all three optimal models (ETa-MEVI2, ETa-NDVI, and ETa-EVI2 (scaled)) underestimated by 14.4%, 10.7%, and 40.7%, respectively, with 28.0%, 16.0%, and 8.0% of days within the tolerance band (Table 3). Overall, the finds showed that ETa-MEVI2 was the top contender for optimal performance in both sites.

In addition to evaluating ETa trends, crop coefficients (Kc) were derived from the ratio of ETa and ETo values for each phase (Table 4). At Site A (irrigated), Kc values were 0.75, 0.76, and 0.66 for S1, S2, and S3, respectively. At Site B (rainfed), Kc values were notably lower, with averages of 0.44 for Phase II and 0.29 for Phase III. The VI-based Kc proxies (NDVI, EVI2 (scaled), EVI2 (Kc), NDVI (scaled), and MEVI2) followed similar trends of in situ Kc across both sites, with generally higher values observed in Site A during peak growth compared to Site B.

### 2.3. Adopting the Optimal Model to Observe the Water Use of Taro Under Different Management Practices

**Figure 3:**
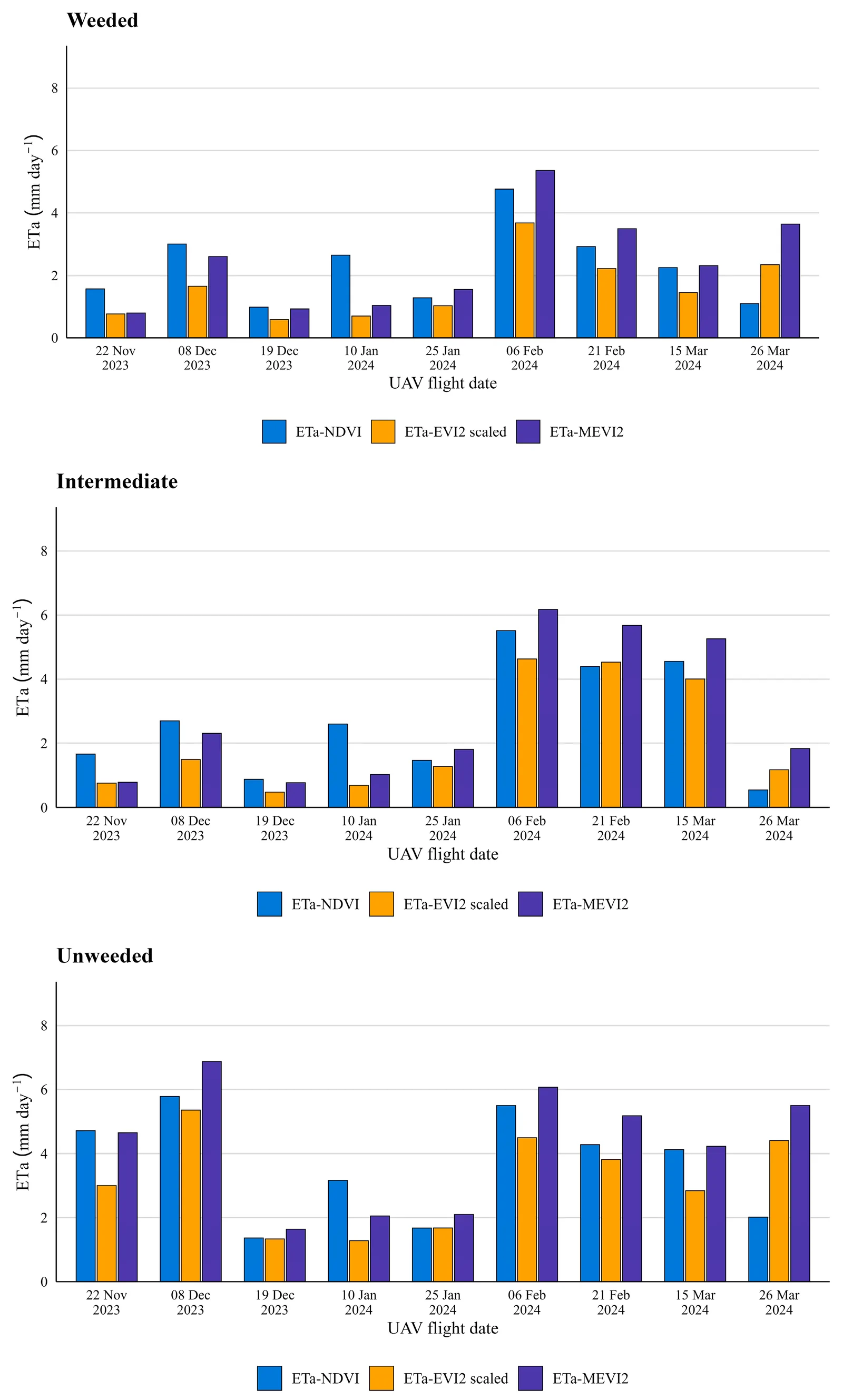
Comparison of optimal VI-based ETa models for rainfed conditions in plots that had different weed management practices on days when UAV imagery was captured.

After validating the VI-based models at the cannabis and taro sites, ETa-MEVI2 was selected as the primary model for application at the rainfed observational field (Site C). Nevertheless, it remained necessary to assess variation in performance among the three optimal models identified at Site B (ETa-MEVI2, ETa-NDVI, and ETa-EVI2 (scaled)). The optimal models were compared using nine UAV images captured throughout the growing season (22 November 2023, 8 December 2023, 6 February 2024, 21 February 2024, 15 March 2024, and 26 March 2024 were captured using the DJI M300, while 19 December 2023, 10 January 2024, and 25 January 2024 were captured using the DJI Mavic 3 multispectral). Based on the results in Figure 3, ETa-EVI2 (scaled) generally had lower ETa estimates than the other models, except at the end of the growing season (26 March 2024). ETa-NDVI had higher ETa estimates during periods of low vegetation cover, which was observed during the first four UAV-imagery dates for the weeded and intermediate fields. ETa-MEVI2 generally had higher ETa estimates during periods of high vegetation cover, and this pattern was observed across all three plots.

**Figure 4:**
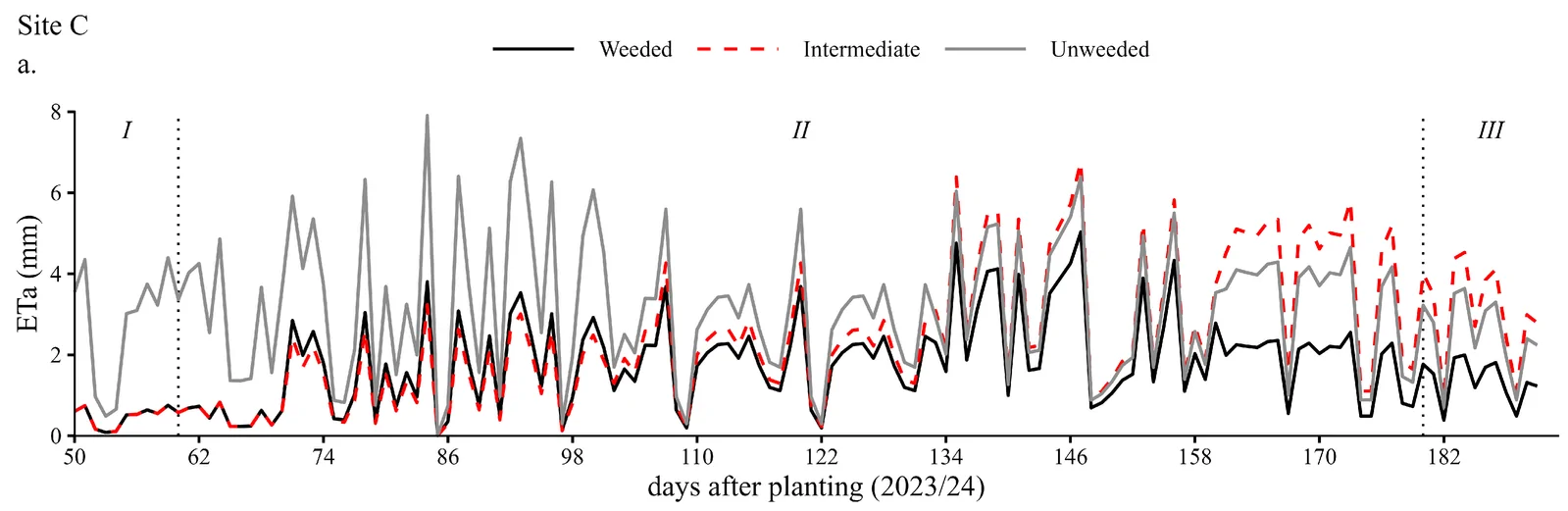

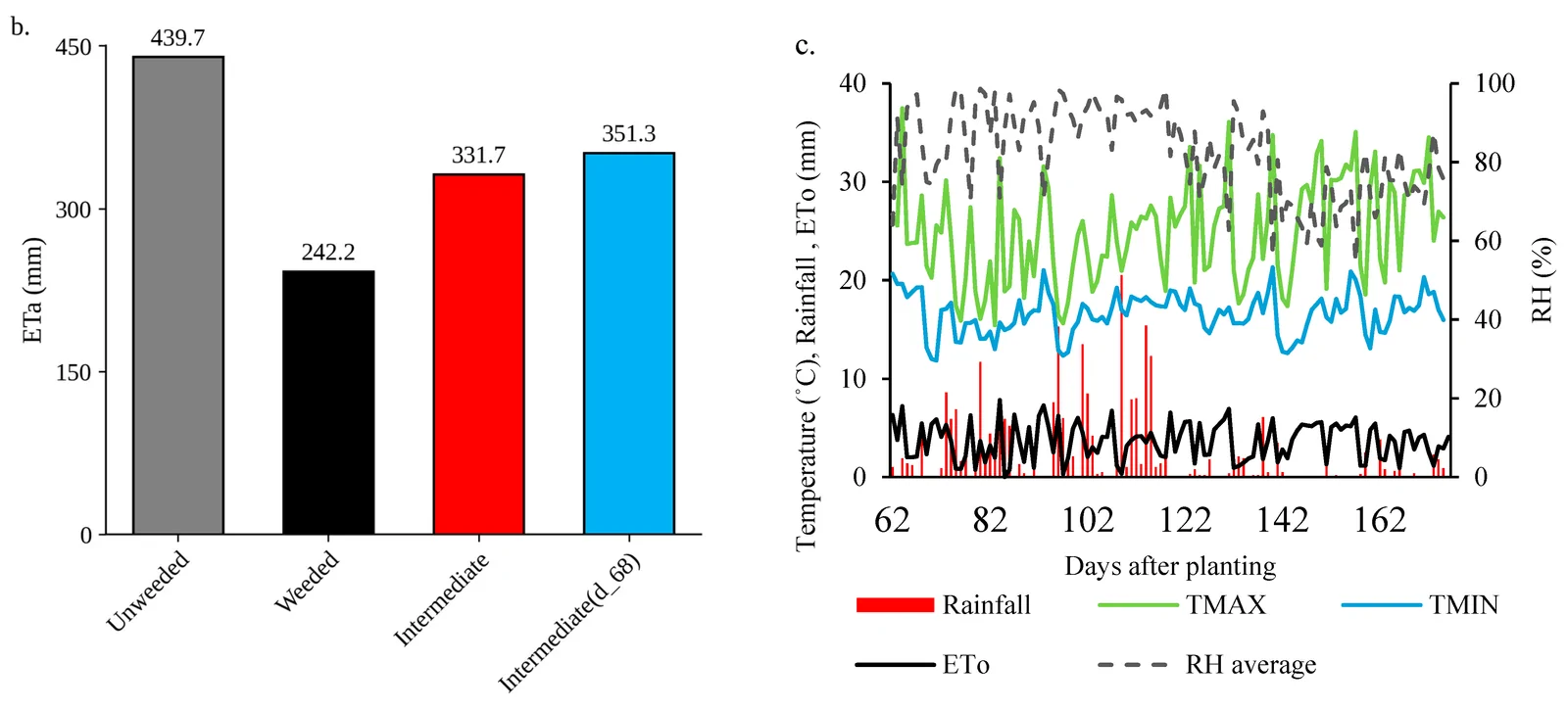
Graphs illustrating (**a**) temporal variability in water use in plots with different weed management practices using an MEVI2-based empirical ETa model throughout the growing season (between day 50 and 191 after planting); (**b**) accumulated ETa derived from the MEVI2-based model for the plots with different weed management practices; (**c**) daily variations in rainfall, reference evapotranspiration (ETo), average relative humidity (RH), and maximum and minimum air temperatures (TMAX and TMIN) from 22 November 2023 to 31 March 2024 in Site C.

ETa-MEVI2 was preferred for the investigation in Site C specifically because it was the most optimal candidate in both validation sites, despite ETa-NDVI showing greater consistency in alignment with measured data for the rainfed taro trial in Site B. Site C consisted of three plots growing taro with different weed management practices (unweeded, weeded, and intermediate). However, the taro in the intermediate field was planted 18 days prior to the weeded and unweeded fields, meaning the three plots were not naturally synchronized in developmental time when data collection began; this planting-date offset is a central condition of the Site C comparison and is addressed explicitly here rather than treated as a minor adjustment. The trial period began after the first round of weeding on 10 November 2023, corresponding to 50 days after sowing for the weeded and unweeded plots (Figure 4a). The intermediate plot was on day 68 at the same time, as its planting had occurred 18 days earlier. To assess whether this offset materially affected the comparison, accumulated ETa was compared between intermediate (d_68) and the hypothetical intermediate field assuming the same timeline as the weeded and unweeded fields; the percentage difference between these two timelines was 5.89%, which was considered small enough to support direct comparison between the plots (Figure 4b), though this remains an assumption of the analysis rather than a demonstrated equivalence.

Daily ETa patterns revealed distinct differences among the management practices (Figure 4a). The weeded field consistently recorded the lowest water use across the observation period, with only a few instances where the intermediate field showed slightly lower values, particularly between 70 and 104 days after planting. This temporary reduction in ETa corresponded with a marked shift in canopy condition (crop yellowing effect due to herbicide application) in the intermediate field. Over the full observation period, the unweeded field accumulated the highest ETa, reaching 449.37 mm, followed by the intermediate (d_68) plot at 351.3 mm and hypothetical intermediate at 331.7 mm. The weeded field accumulated the lowest total ETa of 242.2 mm (Figure 4b). The unweeded plot therefore used 35.5% more water than the intermediate field, while the weeded plot used 27.0% less than the same intermediate reference.

Climatic conditions during the observation period (Figure 4c) contributed to fluctuations in ETa. Rainfall occurred in intermittent pulses, with peak events coinciding with short-term increases in ETa across all plots. Maximum temperatures (TMAX) ranged between 25 °C and 35 °C, while minimum temperatures (TMIN) remained relatively stable between 10 °C and 15 °C. Relative humidity (RH) averaged above 60% throughout the trial, with occasional peaks above 80%. These environmental conditions provided sufficient variability to capture distinct crop water use responses across the management practices. Overall, the results observed associations between differences in ETa patterns and the three management strategies, with the unweeded field showing consistently greater water consumption compared to the weeded and intermediate plots.

**Figure 5:**
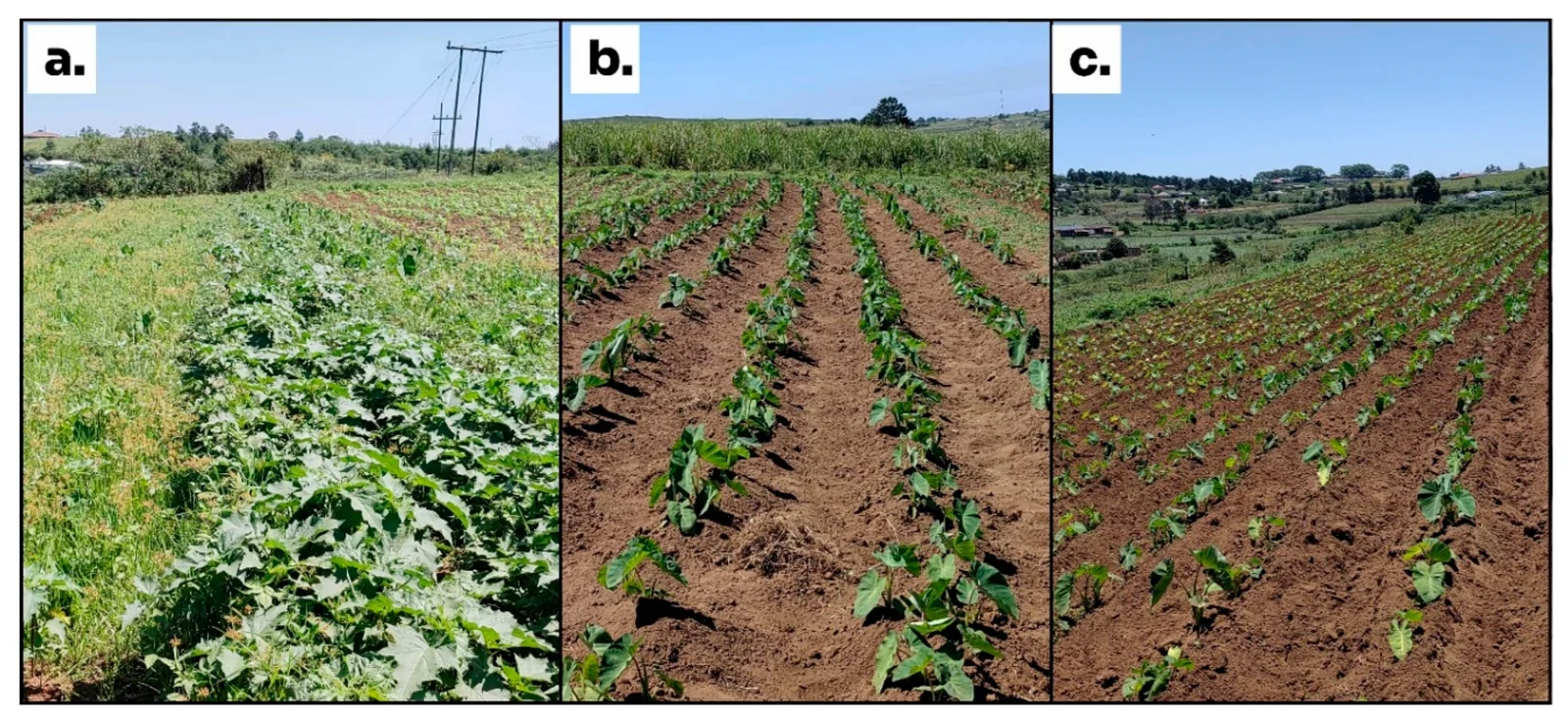
Difference in crop growth on 22 November 2023, under different management practices: (**a**) unweeded (62 DAP), (**b**) weeded (62 DAP), (**c**) intermediate (80 DAP).

**Figure 6:**
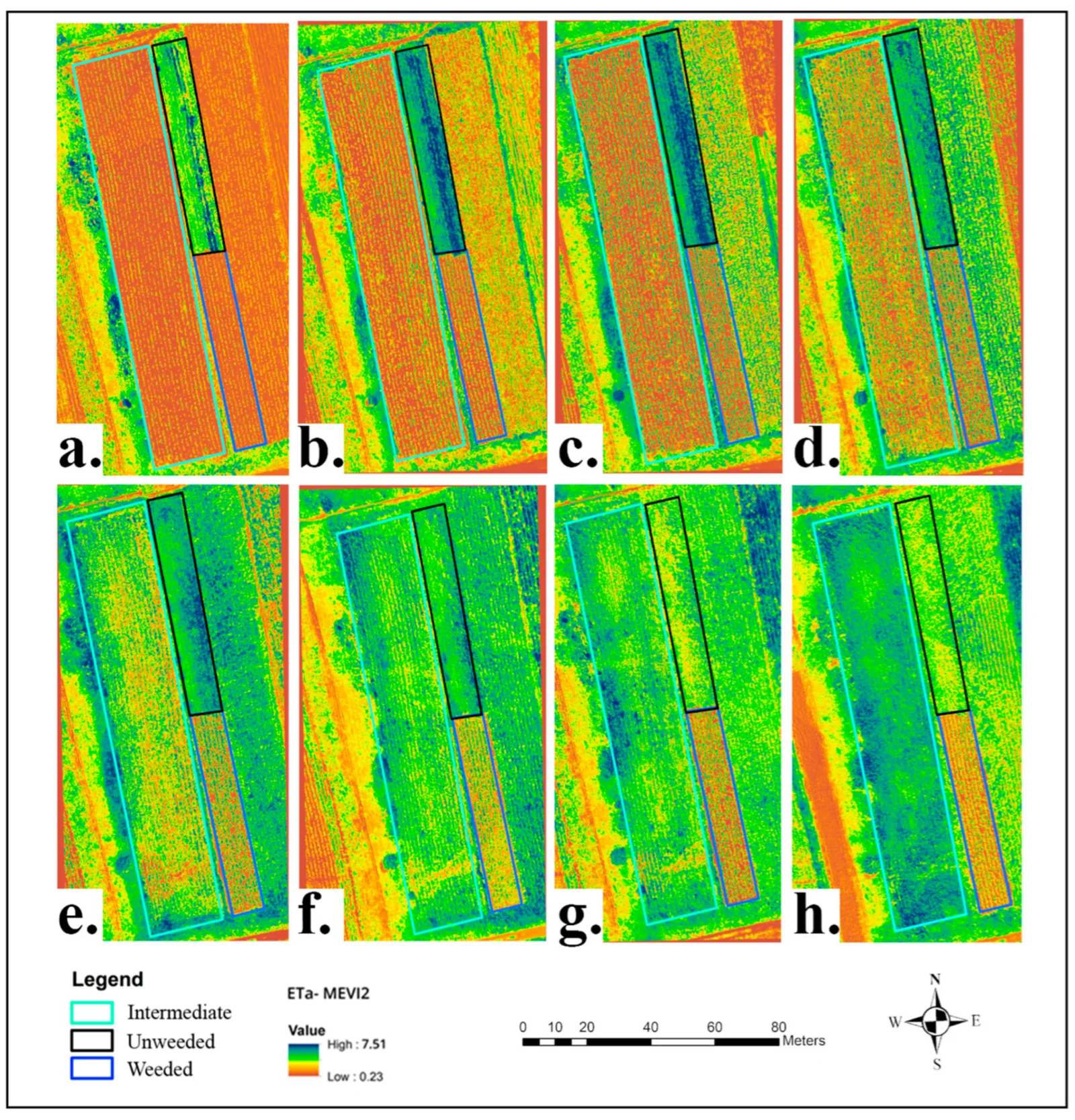
MEVI2-based ETa model maps illustrating the spatio-temporal variation in crop water use across plots with different weed management practices: (**a**) 22 November 2023 (62 DAP); (**b**) 8 December 2023 (78 DAP); (**c**) 19 December 2023 (89 DAP); (**d**) 10 January 2024 (111 DAP); (**e**) 25 January 2024 (126 DAP); (**f**) 6 February 2024 (138 DAP); (**g**) 21 February 2024 (153 DAP); (**h**) 15 March 2024 (175 DAP). The image corresponds with the information in Table 5.

The generation of ETa maps is an essential aspect of effective dissemination of knowledge on observed findings in a way that is simple enough for a person without a scientific background to understand. The ETa trends for the three plots in Site C were visualized (see Figure 5 and Figure 6). These maps were able to capture in detail the changes that took place throughout the observed period. Each image displayed the factors that contributed to the average value calculated using the VI-based empirical model. For example, according to the maps, the area with broad-leaved weeds in the unweeded field (as shown in Figure 5a) could be identified in Figure 6a. Based on observed spatial association, greater vegetation density contributed to higher ETa estimations for a longer period compared to the other parts of that field. Another observation was the influence of erosion on ETa in the intermediate plot. The impact was more apparent in Figure 6e. The advantage of obtaining this data using multispectral UAV imagery with high spatial resolution lies in its ability to provide more insight with precision over time. These changes can be observed by generating land classification maps from the UAV imagery and overlaying them with the simulated Eta, simplifying the process of deducing contributions from different classes. The information presented in Table 5 gave context to Figure 6, regarding the ETo and corresponding MEVI2 (Kc proxy) values on the date of image acquisition.

Based on the crop development stages outlined in Table 12, three growth phases (phases I, II, and III) were observed in this trial at site C, as shown in Table 6, despite only 9 days being captured in phase I, and 11 days in phase III. This information was able to provide MEVI2 (Kc proxy) values presented in Table 7. Some of these values were comparable with the validated data for phases II in Site B (Table 4). According to the captured results for phase II, sites B and C (weeded field) had MEVI2 (Kc proxy) values of 0.50 and 0.51. This was a positive indication, considering that a large portion of both datasets were captured during this grand growth phase where maximum rooting depth and canopy cover occur, and both plots were well-managed. The level of consistency suggests the potential transferability of the model output under similar conditions.

The MEVI2 (Kc proxy) had lower estimates in phase III than phase II for Site B (0.26) and C unweeded (0.77) and weeded fields (0.42), which is expected when vegetation has reached the maturation stage. However, it is important to note that the low Kc value of 0.29 during phase III in Site B not only highlights the fact that the crop had reached its senescence stage, which generally slows down the crop growth rate. This value also indicates potential low ETa estimates induced by seasonal variability. In this instance, Kc value was based on data measured in June, during the winter season in KwaZulu-Natal. Another important factor to note is ETa in the unweeded plot represents combined surface evapotranspiration from taro, weeds, and soil rather than taro water use alone. Further studies should focus on ensuring that more data is captured in phases I and III, assessing the potential impact of other management practices in areas that have different field characteristics and climatic conditions.

### 2.4. Assessing the Cost and Benefit of Adopting These Weed Management Practices

**Figure 7:**
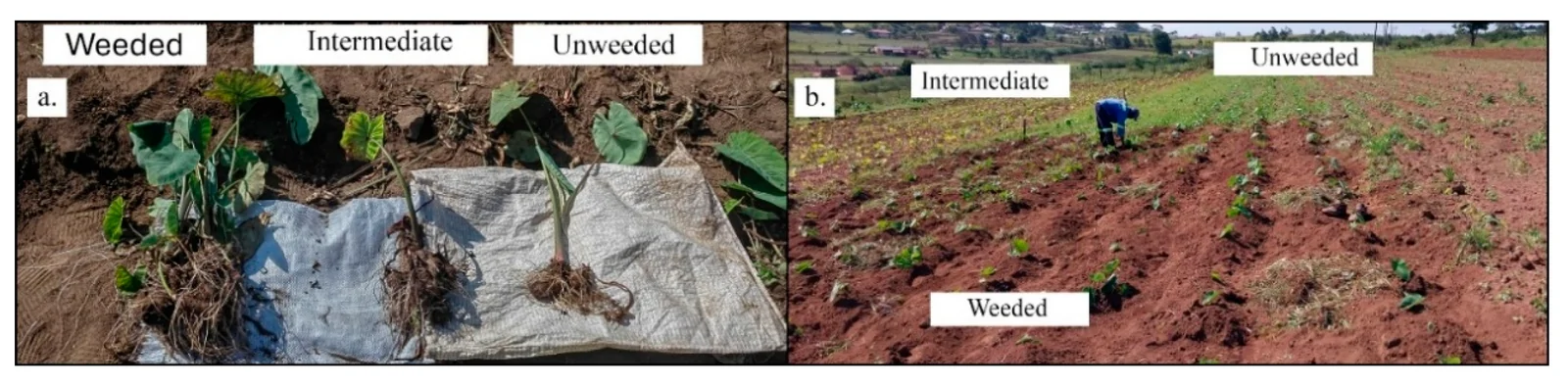
Images illustrating (**a**) the development of taro under different management practices and (**b**) the specific taro plots included in the investigation.

This section aimed to evaluate the practicality of implementing these strategies. It is well established that land management significantly influences crop development. When taro received adequate water, sunlight, and nutrients, its health was physically manifested by maximum root depth, as well as increased number and size of leaves and corms, as shown in Figure 7. Table 8 summarizes the inputs required for each management strategy. After determining the management expenses and revenue generated, the unweeded, weeded, and intermediate fields had a net profit of ZAR 3083 (USD 171), ZAR 16339 (USD 908), and ZAR 13639 (USD 758) (Table 8, Table 9 and Table 10). Weeding produced ZAR 2700 more than the profit generated by the intermediate field, which translates to 19.82%. WUE estimates showed an inverse relationship with weed pressure. The weeded plot was 1.77 (76.5%) times more water-productive than the intermediate field (14.00 vs. 7.93 kg/mm), while the unweeded was 0.15 (−85.4%) time less water productive than the intermediate field (Table 10).

## 3. Discussion

### 3.1. UAV Potential in Smallholder Farms

The use of multispectral UAVs has demonstrated the capability of providing high-resolution spatio-temporal ETa, which can enhance crop management practices at local spatial scales [29]. Although the range covered by UAVs is limited to smaller areas compared to open-source satellites, their ability to acquire accurate data with minimal influence from atmospheric factors also creates the opportunity to enhance our understanding of land cover effects on crop water use in resource-limited regions [30]. In an era where climate change is among the key drivers of sustainable innovations, these platforms can be a great resource for extracting valuable information on NUCs that are under-researched [31].

Although both EC and UAV-based approaches provide valuable insights into crop water use, several factors can contribute to inaccuracies in ETa observations. For EC systems, environmental conditions such as high relative humidity (>70%), low wind speeds, and stable atmospheric conditions suppress turbulence, reducing flux detection and leading to underestimation of ETa [32]. Sensor interference caused by dew, rainfall, or dust deposition on the gas analyzer can further distort readings [33].

For UAV-based multispectral imagery, inaccuracies may arise from both technical and environmental sources. Image quality is highly sensitive to illumination, shadowing, and atmospheric conditions at the time of flight. Variability in solar angle, sensor calibration, or reflectance distortions due to soil background and canopy structure can alter vegetation index values [34]. Flight timing also plays a role, as UAV surveys conducted under variable cloud cover or following rainfall may not capture true canopy conditions [35]. These factors highlight the importance of calibration, frequent acquisition of imagery (to accurately depict the fluctuations in meteorological conditions), and validation.

In addition to acquisition-related uncertainty, the gap-filling used to estimate missing observation dates can itself introduce errors that can affect model performance, as seen between Section 2.1 and Section 2.2 where ETa-NDVI ranked third at both Site A and B after validating the days UAV images were captured, but changed to fourth and second, respectively, when gap-filling using monthly Kc proxy composites was introduced. Although there is no independent analysis on the effect of gap-filling, its potential influence must be acknowledged. Capturing more images during the growing season reduces the composite timestamp (contributing towards a better representation of daily variations) and allows evaluation of the impact of gap-filling on the discrepancies between observed and simulated data [36].

### 3.2. Evaluation of VI-Based ETa Models

The validated data from this study identified ETa-MEVI2, ETa- NDVI, and ETa-EVI2 (scaled) as optimal models for estimating ETa for taro under rainfed conditions. While NDVI has traditionally been widely used, its limitations became evident under dense canopy or irrigated conditions. Despite ETa-NDVI being among the suitable models for observing taro water use, no studies support it as the best-performing model except for Abbasi et al. [37], where the model accurately estimated the long-term annual average ETa of wheat. According to D’Urso [38], ETa-NDVI will always have lower estimates, under well-watered and unstressed conditions, because Kc maximizes at 1.2 while the empirical model does the same at 1 (the model does not account for this parameterization). This pattern, where ETa-NDVI had the lowest performance, was observed in Site A, which grew irrigated cannabis. Jarchow et al. [39] highlighted that the NDVI is sensitive to imagery that does not have maximum vegetation cover. This could be the reason that best explains why the ETa-NDVI model was more consistent with measured data in the rainfed taro field (Site B). One of the main limitations of this model is its suitability in unstable conditions and unreliability in extreme events such as disease outbreaks and floods [40].

ETa-EVI2 (Kc) was the superior model in a sugarcane water use study conducted by Woldemariam et al. [24]. Similar findings were observed in Site A, which suggests that the EVI2-based models are more sensitive to irrigated crops or those with greater canopy density. In Site B, EVI2-based models recorded higher values during the peak growing season. Findings from other studies that adopted the non-modified version of ETa-MEVI2 also noted similar performances when compared to the NDVI-based models [37,41,42]. The ETa-MEVI2 underestimated towards the end of the season, and this could be attributed to the decrease in plant available water due to changes in seasons (winter), which often causes a decrease in LAI (LAI is directly proportional to EVI2 when using Beer–Lambert’s equation), to match environmental conditions that enable photosynthesis [43]. The opposite was observed in Site A, where ETa-MEVI2 overestimated towards the end of the growth cycle of cannabis. Nouri et al. [42] confirmed that EVI2 has a greater sensitivity to crop structure and greenness compared to NDVI.

In summary, ETa-MEVI2 is the optimal model capability of capturing better results in both rainfed and irrigated regimes. ETa-NDVI was a better contender under sparse or rainfed conditions, while ETa-MEVI2 outperformed it under dense canopy or irrigated systems due to their structural resilience to saturation and soil background effects. This pattern was observed when optimal models were compared in Site C (Figure 3). These insights emphasize the importance of index selection based on vegetation dynamics and environmental conditions, especially for developing context-specific ETa models in smallholder systems.

The use of plant characteristics and environmental factors as proxies to understand the development of a crop holds significant potential in an era where deep learning tools are becoming more advanced. Atanasov [44] evaluated the potential of tracking the RGB color of tomato leaves as an indicator of water status. Prior to these findings, their research had established a relationship that could predict soil moisture from RGB and temperature [45]. This demonstrates that there are other affordable in situ methods that can serve as complementary approaches for gathering more information on NUCs.

### 3.3. Contribution of Findings in Understanding Taro

This study demonstrated the potential of empirical ETa models derived from multispectral UAV imagery for monitoring crop water use in smallholder farms. The findings provided insight into the sensitivity of different VIs to crop–environment interactions, especially under varying water management conditions. While these tools proved to be effective based on the observational study in Site C, it is important to note that one of the major limitations of this study was the lack of replication in the treatments for the different management practices. As a result, it cannot be stated with absolute certainty that the outcomes were due to the weed management measures alone. Future research should ensure that this limitation is addressed in experimental design. The proxies were able to capture Kc values across different growth phases and management practices, demonstrating their potential as an effective tool for refining parameters used to identify suitable areas.

Mengistu et al. [46] investigated the water use of taro in the Mbongolwane wetland (approximately 40 km west of Eshowe, KwaZulu-Natal, South Africa) and reported an average Kc value of 0.6. Fares [47] found Kc values of 1.05, 1.15, and 1.1 for the early, mid, and late stages of taro under flood irrigation in Hawaii. These comparative values demonstrate the variability in water requirements across environments and irrigation regimes. Although NUCs are severely under-researched, there is evidence supporting the contribution of environmental effects on their development. Mabhaudhi et al. [1] conducted a study that observed taro’s response to varying water regimes (30%, 60%, 100%) in Pretoria, South Africa. Findings from the study demonstrated a direct correlation between the unstressed and well-watered taro (100%) and higher yield, canopy size, and plant height. These findings were consistent with observations in this study. The taro in the unweeded field had less access to water, resulting in lower yield, canopy size, and plant height, which is evident in Figure 7. Overall, higher water availability (100%) led to increased yield, larger canopy size, and greater plant height trends that were consistent with the well-managed taro plots observed in this study.

Importantly, this study also highlights an observed association between high vegetation density and ETa. In the unweeded field, the presence of unmanaged weeds coincided with greater total vegetative cover, which was associated with higher estimated evapotranspiration, plausibly reflecting additional transpiring surfaces and interspecies competition for water. This pattern co-occurred with lower crop vigor, smaller canopy size, and reduced yield in the same field. It is plausible that weeds reduce stomatal conductance in taro by intensifying water stress, thereby diminishing both transpiration and photosynthetic activity [48,49]; however, this physiological pathway was not directly measured in this study and remains an inference rather than an observed outcome. These findings underscore the critical need for effective weed management, not only to improve crop productivity but also to optimize water use efficiency in water-scarce environments. The improved water use efficiency in the weeded and intermediate plots translated to improved economic benefits (cost benefit analysis). This finding agreed with results from Fadlallah et al. [50], on work conducted with herbicides in Egypt.

Although the weeded field generated a substantial amount of money through higher yields, some factors need to be considered before adopting the practice. This approach has the potential to keep people employed during the growing season, but in many African countries, some cannot afford to pay for the work. The practice also contradicts the guidelines set by conservation agriculture, which are against tilling. Constant tilling has been found to degrade soil quality and increase the potential of becoming more prone to erosion [51]. Over time, the loss of organic matter through this process can cause a reduction in soil fertility, resulting in potentially low yield turnover. Conservation agriculture researchers have encouraged farmers to adopt integrated weed management to preserve the soil [52]. To some degree, methods applied in the intermediate field align with conservation agriculture guidelines [53]. The farmer demonstrated an understanding of maintaining the soil quality by integrating weed management strategies and ensuring they obtain reasonable yields with low input while minimizing tilling.

## 4. Materials and Methods

### 4.1. Study Site Description

**Figure 8:**
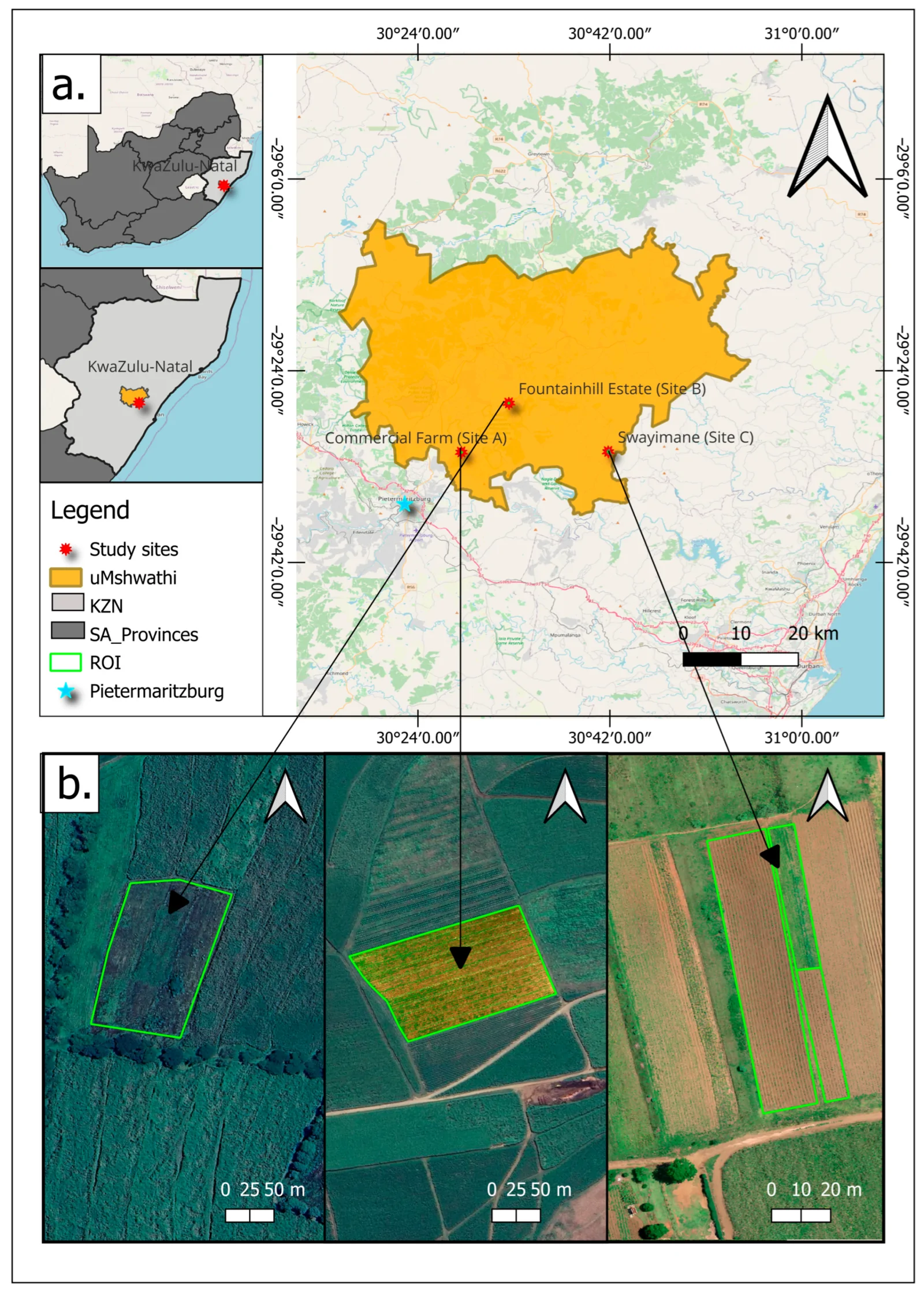
Map showing (**a**) the locations of the areas of interest within the uMshwathi local municipality, KwaZulu-Natal, South Africa; (**b**) the boundary demarcations of the study sites. RGB UAV imagery was captured for the Commercial Farm (Site A) on 18 January 2023, Fountainhill Estate (Site B) on 6 June 2022, and Swayimane (Site C) on 22 November 2023.

The study was conducted across three sites within the uMshwathi Local Municipality, KwaZulu-Natal Province, South Africa (Figure 8): Site A, a commercial farm approximately 15 km northeast of Pietermaritzburg (29°31′38.7″ S; 30°28′4.3″ E; 812 m a.s.l.); Site B, Fountainhill Estate (29°26′56.83″ S; 30°32′48.80″ E; 905 m a.s.l.); and Site C, Swayimane (29°31′33.2″ S; 30°41′51.8″ E; 840 m a.s.l.). Sites A and B served as model-testing sites, used to evaluate and select among candidate VI-based ETa models against in situ eddy covariance data, while Site C was the site of the final application, in which the selected model was used to compare taro water use under different weed management practices. The area is primarily used for agriculture, with sugarcane, maize, taro, and sweet potatoes being the main crops grown. Much of the farming in the region relies on summer rainfall, which typically occurs between October and February. Various studies conducted within the local municipality have reported that the area experiences average minimum and maximum temperatures ranging between 11 °C and 24 °C (which correlates with the measurement meteorological data shown in Table 11) and mean annual precipitation ranging between 800 and 1200 mm [54,55,56].

### 4.2. Field Design

#### 4.2.1. Site A (Commercial Farm)

The study site covered an area of approximately 25,880 m^2^, and the crop that was being observed was cannabis (*C. sativa* L). The growing season for this trial was approximately 5 months between November 2022 and April 2023. Drip irrigation was implemented to ensure the plants had sufficient water available for optimal growth during periods of low rainfall. The fertilizer was dissolved for application using the same system. This irrigation system promotes efficient water use and better nutrient uptake, thus aiding in the sustainable use of resources while maximizing productivity. Manual labor was used in removing weeds, setting up the irrigation network, and during the harvesting period. The inter-row spacing was 2.5 m, while the intra-row spacing (space between plants in a row) was 2 m, respectively. The texture of the soil samples was characterized as sandy clay loam (74% sand, 5% silt, and 21% clay at a depth between 0 and 0.2 m below the topsoil surface, with a pH between 4.12 and 5.54). The topography of the region of interest (ROI) had a gentle slope of 13.1%. The data for this trial were from a study conducted by Denton et al. [57]. The growth phases used in this study were based on information in Table 12.

#### 4.2.2. Site B (Fountainhill Estate)

The study site was demarcated to cover an area of approximately 9750 m^2^, and the crop being investigated in this trial was taro (landrace Dumbe-dumbe). The inter-row spacing was 1 m, while the intra-row spacing was 0.5 m. The duration of this experiment was between December 2021 and June 2022. This trial was purely rainfed. Workers from the nearest community (Swayimane) were contracted to assist with planting, weed management, application of fertilizer (gromo accelerator), herbicide (gramoxone), and harvesting. The soil texture for this area was characterized as sandy loam (80% sand, 4% silt, and 16% clay, at a depth ranging between 0 and 0.3 m). The data for this trial were from a study conducted by Kunz et al. [59].

#### 4.2.3. Site C (Swayimane)

**Figure 9:**
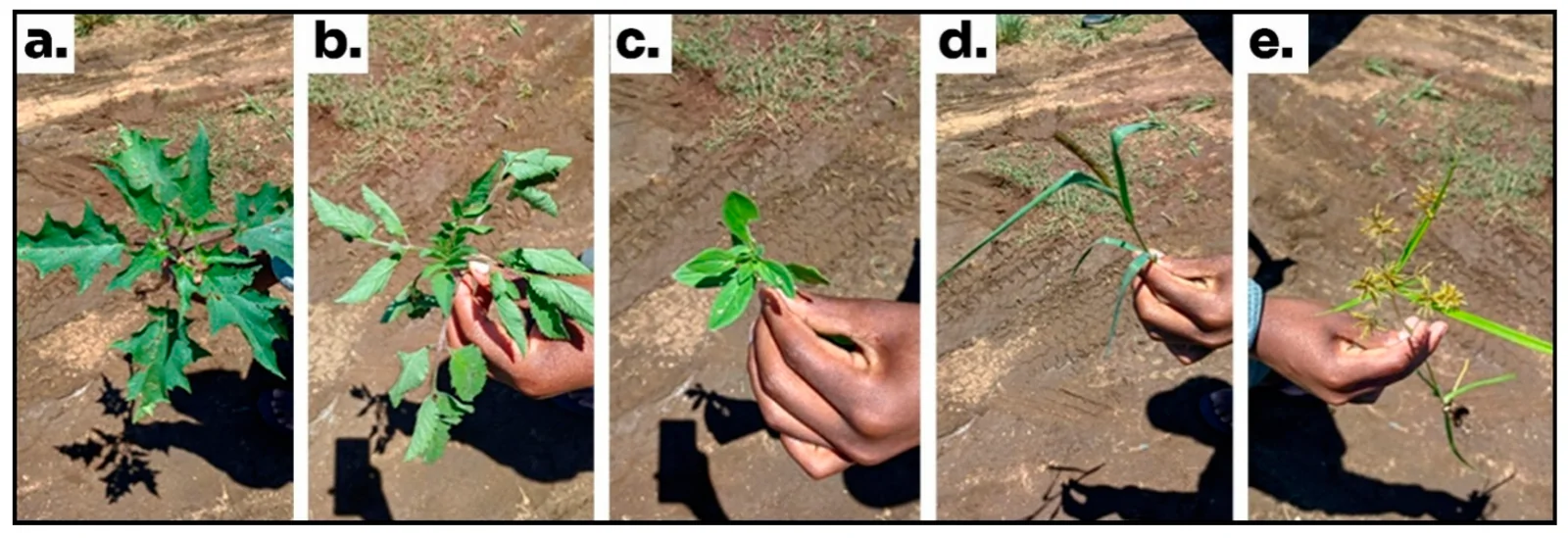
Types of weeds identified within the boundary of the taro field at the study site: (**a**) Jimsonweed (*Datura stramonium* L.), (**b**) Blackjack (*Bidens pilosa*), (**c**) Spermacoce, (**d**) Wheat grass (*Poaceae*), and (**e**) Nut grass (*Cyperus rotundus*). Images were captured on 8 December 2023.

A workflow that could be adopted to observe the impact of different weed management practices on the water use and yield of taro was investigated in Site C as an observational field comparison. Initially, the study site was demarcated to cover an area of 980 m^2^. The proposed field was designed to consist of 2 split plots, where one was weeded throughout the growing season, while the other was unweeded. After the bulbs were sown, we were notified that the farmer had planted the same crop in the field adjacent to our study area. This new field was added as the third plot to enhance the study by gaining insight into the effectiveness of current management practices used by the farmer. Thus, the study area was increased to 2276 m^2^. The third plot was identified as the intermediate field because the farmer applied conventional weed management strategies. These include cultivating using cattle, applying herbicide (farmag paraquat 200), and manual labor during the first 3 months after planting. The soil texture for the ROI was characterized as sandy loam (75% sand, 7% silt, and 18% clay, at a depth ranging between 0 and 0.4 m). The study site had five species identified as weeds in the field (Figure 9).

It is important to note that manure was applied in all three plots. The inter- and intra-row spacing was the same as Site B (1 m and 0.5 m, respectively). However, the planting dates were different. The taro bulbs in the intermediate field were sown on 4 September 2023, while the bulbs in the weeded and unweeded fields were sown on 22 September 2023. The first round of the weeding took place, on 10 November 2023, for the weeded field. This process was repeated every fortnight. Harvesting took place in early April 2024. The growth phases used in this study were based on information in Table 13.

### 4.3. Data Acquisition

#### 4.3.1. In Situ ETa Data Acquisition and Preprocessing

**Figure 10:**
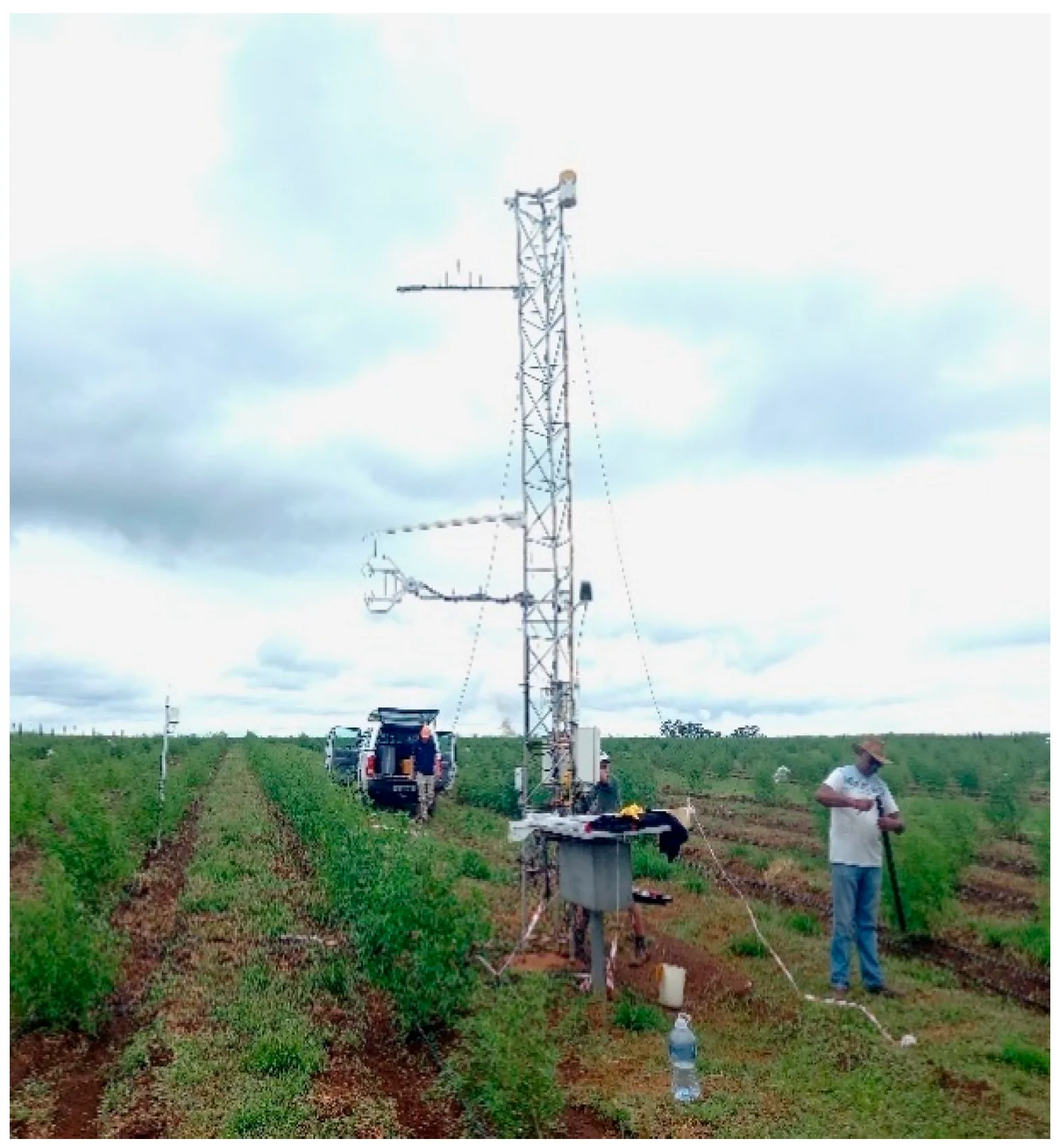
Installation of the eddy covariance flux tower at Site A.

**Figure 11:**
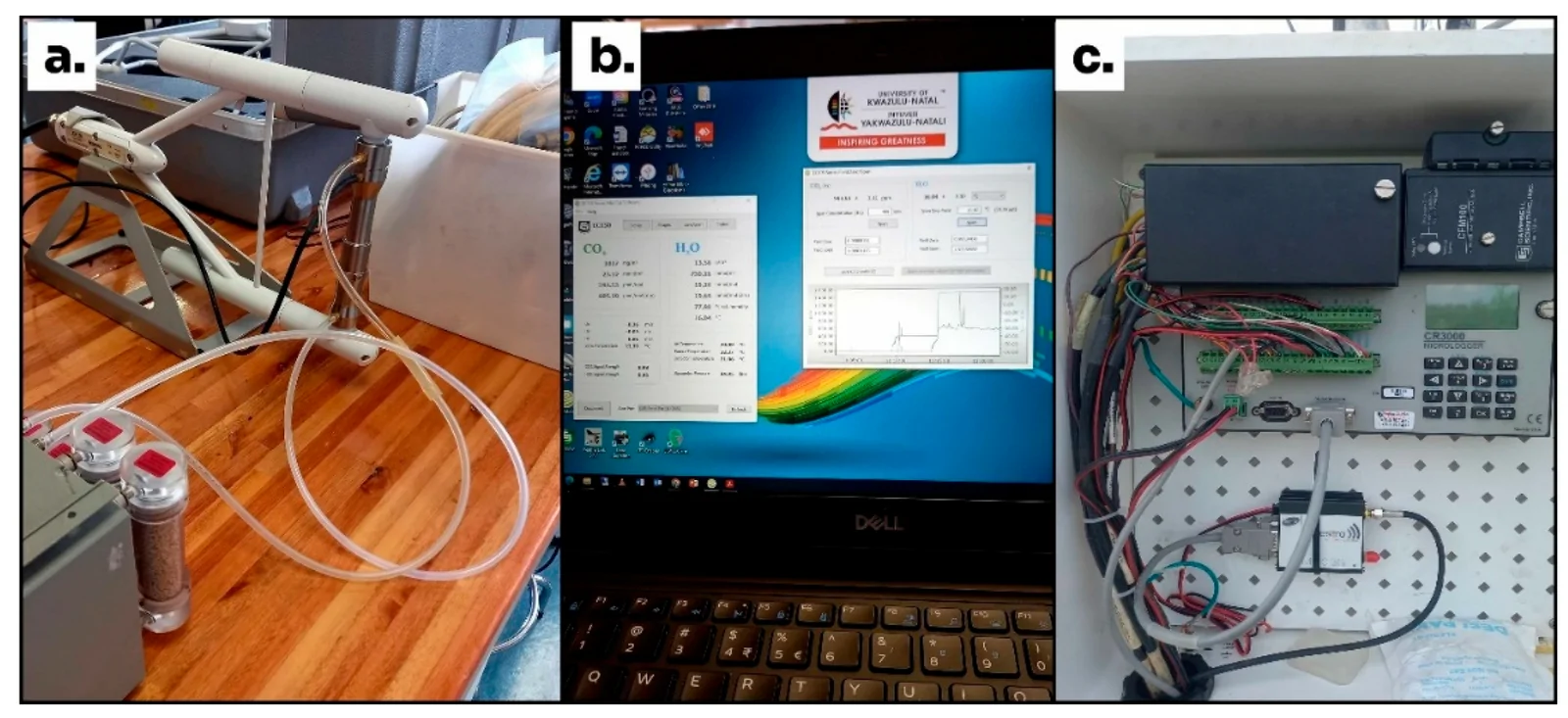
Calibration process components: (**a**) EC150 CO_2_/H_2_O Open-Path Gas Analyser, (**b**) configuration using the EC100 Series Monitor software, and (**c**) the Campbell CR3000 datalogger in the field after all sensors were connected.

An eddy covariance system was set up to monitor sites A and B’s microclimatic conditions and crop water use. The flux tower comprised multiple sensors (as depicted in Figure 10 and listed in Table 14) that were connected to a Campbell CR3000 datalogger programmed to measure data at a frequency of 10 Hz, which was averaged at 30 min intervals. All the components on the flux tower were powered by two 12 V direct current batteries connected to a solar panel that was mounted on the strong box (Figure 10 and Figure 11). Several factors had to be considered when deciding where the EC system was mounted in the respective fields. These included topography, flux footprint, and prevailing wind direction (the rule of thumb proposes that 1 m above the crop canopy can provide a fetch of approximately 100 m). Failure to adhere to these guidelines can result in inaccurate in situ measurements. The flux tower captures ETa data in two ways (direct and indirect). This study relied on ETa data captured using the direct approach. This method uses the sonic anemometer (CSAT3A, Campbell Scientific, Logan, UT, USA) and the open path gas analyzer (EC150, Campbell Scientific, Logan, UT, USA) functioning simultaneously to measure the concentration of moisture content, also referred to as latent heat flux (λET) (W.m^−2^) in the atmosphere within the footprint range. In sites A and B, these sensors were mounted at 2 m height and 1.5 m above the crop canopy. Adjustments were made throughout the growing season to ensure the height above the crop was constant in both fields. Furthermore, λET was determined utilizing Equation (1).

(1) $$\lambda ET=L_{v}\cdot \bar{w^{\prime }{\rho^{\prime }}_{w}}$$

where λET (W.m^2^) was calculated using latent heat of vaporization (L_v_ = 2.45 * 10^6^ J.kg^−1^, at 20°C), and the mean covariance of fluctuation in vertical wind speed (w), and fluctuation in water vapor density (ρ_w_) (kg.m^−3^). The indirect method calculates λET as a residual of the energy balance model using Equation (2). Net radiation (Rn) was measured using the CNR4 Net Radiometer (Kipp and Zonen, Delft, The Netherlands). The net radiometer was installed at a height of approximately 3 m above the crop canopy in both sites. Soil water content sensors (CS616 Water Content Reflectometers, Campbell Scientific, Logan, UT, USA) were installed at different depths [in Site A (0.2 m, 0.4 m, and 0.6 m), and B (0.15 m, 0.3 m, and 0.6 m)], and soil temperature thermocouples (TCAV Type E thermocouples, Campbell Scientific, Logan, UT, USA) were deployed to determine soil heat flux (G) [in Site A and B (0.02 m, 0.05 m, and 0,08 m)]. Sensible heat flux density was measured using the sonic anemometer [61].

(2) λET=Rn−G−H

where λET (W.m^−2^) was determined as the difference between net radiation (Rn) (W.m^−2^), soil heat flux (G) (W.m^−2^), and sensible heat flux (H) (W.m^−2^). Daily ETa was obtained using Equation (3), and the 86,400 represents the number of seconds in 24 h.

(3) $$ETa=\frac{\lambda ET}{L_{v}}*86,400$$

The remaining sensors recorded microclimate variables used for short-grass daily reference evapotranspiration (ETo) calculations via the FAO-56 Penman–Monteith method (Equation (4)). Two reference surfaces are typically applied: short grass (0.12 m height) and alfalfa (0.5 m height), with the former generally used for low vegetation and the latter for taller crops [29,61]. The calculation assumes that the reference crop is well-watered and not under stress. Under these conditions, ETo is estimated using the following equation:

(4) $$ETo=\frac{0.408\Delta (Rn-G)+\gamma (\frac{900}{T+273})u_{2}(e_{s}-e_{a})}{\Delta +\gamma (1+0.34u_{2})}$$

where reference evapotranspiration (ETo) (W.m^−2^) was determined using the following variables: the slope of the saturation vapor pressure curve (Δ), net radiation at the crop surface (Rn) (W.m^−2^), soil heat flux (G) (W.m^−2^), psychrometric constant (γ), mean daily air temperature at 2 m height (T) (°C), wind speed at 2 m (u_2_) (m.s^−1^), saturation vapor pressure (e_s_) (pascals), actual vapor pressure (e_a_) (pascals), vapor pressure deficit (VPD) (e_s_−e_a_) (pascals).

When ETa and ETo have been determined, the crop coefficient (Kc) can be calculated by using Equation (5) below. To obtain Kc on a daily or monthly temporal scale, it is essential to ensure that the other variables (ETa and ETo) also use the same timestamp.

(5) $$Kc=\frac{ETa}{ETo}$$

To ensure the reliability of the dataset and minimize the influence of errors from malfunctioning sensors, several quality control measures were implemented. The steps outlined below were followed to improve data quality and ensure that only accurate and relevant information was used in the analysis:

- Data Filtering: Only the 30 min data recorded between 07:00 and 17:00 SAST were stored for ETa estimation because it represented a period when evapotranspiration normally occurs. Low turbulence normally occurs at night.
- An internet modem was installed on the eddy covariance flux tower to ensure that regular updates were uploaded to an online server. This allowed the early detection of malfunctioning sensors or low battery voltage, which significantly minimized data loss (less than 7%). If the Kc value could not be obtained using residual ETa (indirect method), the average Kc value from the growth phases estimates would be obtained.
- Negative λET values were omitted, since they indicated potential malfunction of sensors that are often caused by interference, exposure to condensation or power shortage. These values were interpolated by calculating the product of daily ETo and the average monthly Kc value.
- Data had to be excluded when Rn was negative or G was greater Rn. In such instances, a correction factor for G had to be applied.
- The energy balance closure was determined in both sites (A and B). The method that was adopted was a ratio between (H + λET) and (Rn–G). A value closer to 1 indicates near-perfect energy balance closure [62]. The two sites had an energy balance closure above 75%.

#### 4.3.2. Remotely Sensed Data Acquisition and Preprocessing

All the remotely sensed data were captured using a Mica Sense Altum (MicaSense, Seattle, WA, USA) camera and a Downwelling Light Sensor 2 (DLS-2) (DLS-2) (MicaSense, Seattle, WA, USA) mounted on a DJI Matrice 300 UAV (DJI, Shenzhen, China) in sites A and B. The same RS platform was used in Site C. However, there were a few instances where the DJI Matrice 300 was not available (in Site C).

The DJI Mavic 3 multispectral (DJI, Shenzhen, China) was employed as an alternative (band specifications presented in Table 15). The images captured by the Mavic 3 were not used for temporal and cumulative ETa estimations but visualizing the different management practices maps only at the time. The Mica Sense Altum camera imagery was captured at a ground sampling distance (GSD) of 0.07 m per pixel, with the UAV flying at an elevation of 100 m above the ground, while the Mavic 3 multispectral captured footage at a GSD of 0.007 m per pixel, flying at an elevation of 15 m above the ground. A keyhole markup language (KML) file containing the demarcations of the ROI was generated using Google Earth Pro (version 7.3.6.9345) and imported into the DJI smart controller for flight planning. The calibration reflectance panel (CRP) was used before each flight to ensure the camera was calibrated according to the lighting conditions on the day. The camera had an 80% frontal overlap, while the side overlap was 70%, to enhance image stitching accuracy that is often affected by geometric distortions resulting from environmental factors such as wind (which can destabilize the sensor) and uneven surface illumination due to terrain and slope. The process also minimizes the likelihood of data gaps occurring during flight.

Remote sensing data were captured every fortnight on days with clear skies between 10:00 a.m. and 12:00 p.m. South African Standard Time (SAST) to ensure consistent lighting conditions. Image correction was performed during pre-processing using Pix4Dfields (version 2.7.2) by selecting settings that account for weather conditions. All images captured on each day were stitched together to form an orthomosaic. The same application was used to generate the ROI shapefiles. The RS dataset used in this study for Site A was captured between January 2023 and April 2023, Site B between February 2022 and June 2022, and site C between November 2023 and March 2024.

All stitched images and shapefiles containing the demarcation of the ROI in all three sites were uploaded to Google Earth Engine (GEE). The image collection was resampled to a spatial resolution of 0.07 m per pixel. A script was generated to extract the NDVI and EVI2 values for water use estimation using empirical models on the platform.

### 4.4. Methodology for Generating VI-Based ETa Empirical Model

In identifying an optimal approach to estimate water use in smallholder farms, resource availability was a key consideration. The multispectral sensors employed in this study lacked specific spectral bands (e.g., those needed to derive albedo, which requires shortwave radiation), limiting their suitability for most surface energy balance methods. This limitation led to the adoption of vegetation index (VI)-based workflows, which are better suited to smallholder contexts due to their lower input requirements [23,24,37,41]. The empirical models developed in this study utilized NDVI and EVI2 as proxies for crop coefficient (Kc). NDVI, a widely used indicator of crop health, captures vegetation greenness by reflecting chlorophyll content and canopy density (Equation (6)) [54].

(6) $$\mathrm{NDVI}=\frac{NIR-RED}{NIR+RED}$$

Some studies highlighted that NDVI is prone to errors in areas with low vegetation cover and high soil reflectance [34,63]. According to Woldemariam et al. [24], EVI2 was developed to address some of the limitations induced by using NDVI (Equation (7)) [42].

(7) $$\mathrm{EVI}2=2.5*\frac{\left(NIR-RED\right)}{NIR+2.4*RED+1}$$

Three empirical formulas were applied to both VIs (NDVI and EVI2). The first ETa estimation directly calculated the selected VIs and ETo, as shown in Equations (8) and (9).

(8) ETa_NDVI=NDVI∗ETo

(9) ETa_EVI2=EVI2∗ETo

The second formula was a scaled version based on the assumption that under well-watered and unstressed conditions, the maximum value for NDVI and EVI2 is 1, while the Kc value is 1.2 [37], as shown by Equations (10) and (11)).

(10) $${NDVI}_{scaled}=1.2*NDVI$$

(11) $${EVI2}_{scaled}=1.2*EVI2$$

The ETa for the scaled VIs was calculated using Equations (12) and (13).

(12) $$ETa\_{NDVI}_{scaled}={NDVI}_{scaled}*ETo$$

(13) $$ETa\_{EVI2}_{scaled}={EVI2}_{scaled}*ETo$$

The third formula observed the linear regression between Kc and the respective VIs at different growth stages based on the assumptions stated by Woldemariam et al., Abbasi et al., Yacoob et al. [24,26,37], as shown in Equations (14) and (15).

(14) $${NDVI}_{Kc}=1.25*NDVI+0.2$$

(15) $${EVI2}_{Kc}=1.25*EVI2+0.2$$

The ETa for the Kc-based empirical formulas was calculated using Equations (16) and (17):

(16) $${ETa\_NDVI}_{Kc}={NDVI}_{Kc}*ETo$$

(17) $${ETa\_EVI2}_{Kc}={EVI2}_{Kc}*ETo$$

The fourth formula was a modified version of EVI2 generated by Abbasi et al. [36] based on findings from Nagler et al. [43] and Abbasi et al. [23], which incorporates the Beer–Lambert law to express canopy light absorption and maximum, as shown in Equation (18).

(18) $$MEVI2=1.5125\left(1-e^{-2.25*EVI2}\right)-0.169$$

The ETa obtained from adopting MEVI2 as a proxy for Kc was calculated using Equation (19).

(19) ETa_MEVI2=MEVI2∗ETo

### 4.5. Assessing the Performance of the VI-Based ET Empirical Model Estimates

To identify the optimal models, all VI-based ETa estimates for the days on which UAV imagery was acquired were validated against in situ daily ETa measurements recorded at Sites A and B. Their performance was assessed based on three statistical metrics: the coefficient of determination (R^2^) (Equation (20)), mean absolute error (MAE) (Equation (21)), and root mean square error (RMSE) (Equation (22)), each assigned a weighting of 0.2, 0.4, and 0.4, respectively, for the comparison. Coefficient of determination was assigned a lower weighting because a high R^2^ correlation does not imply low error magnitude [64].

(20) $$R^{2}=1-\frac{{\sum_{i=1}^{n}(y_{i}-{\hat{y}}_{i})}^{2}}{{\sum_{i=1}^{n}(y_{i}-{\bar{y}}_{i})}^{2}}$$

(21) $$MAE=\frac{\sum_{i=1}^{n}⃒y_{i}-{\hat{y}}_{i}⃒}{n}$$

(22) $$RMSE=\sqrt{\frac{{\sum_{i=1}^{n}(y_{i}-{\hat{y}}_{i})}^{2}}{n}}$$

To observe the performance of each model throughout the growing season, monthly composites from the VI-based Kc proxies (averages from the images captured in a month) were multiplied by daily ETo to obtain daily ETa. Temporal, violin plots and cumulative graphs were generated for comparison in both sites. The temporal graph had a ±20% tolerance band for the measured data to identify models that were within the acceptable error limit throughout the growing season [65]. The observations from the validated sites enabled selection of optimal models for assessing ETa site C.

### 4.6. Assessing the Cost and Benefit of Implementing Different Weed Management Practices

**Figure 12:**
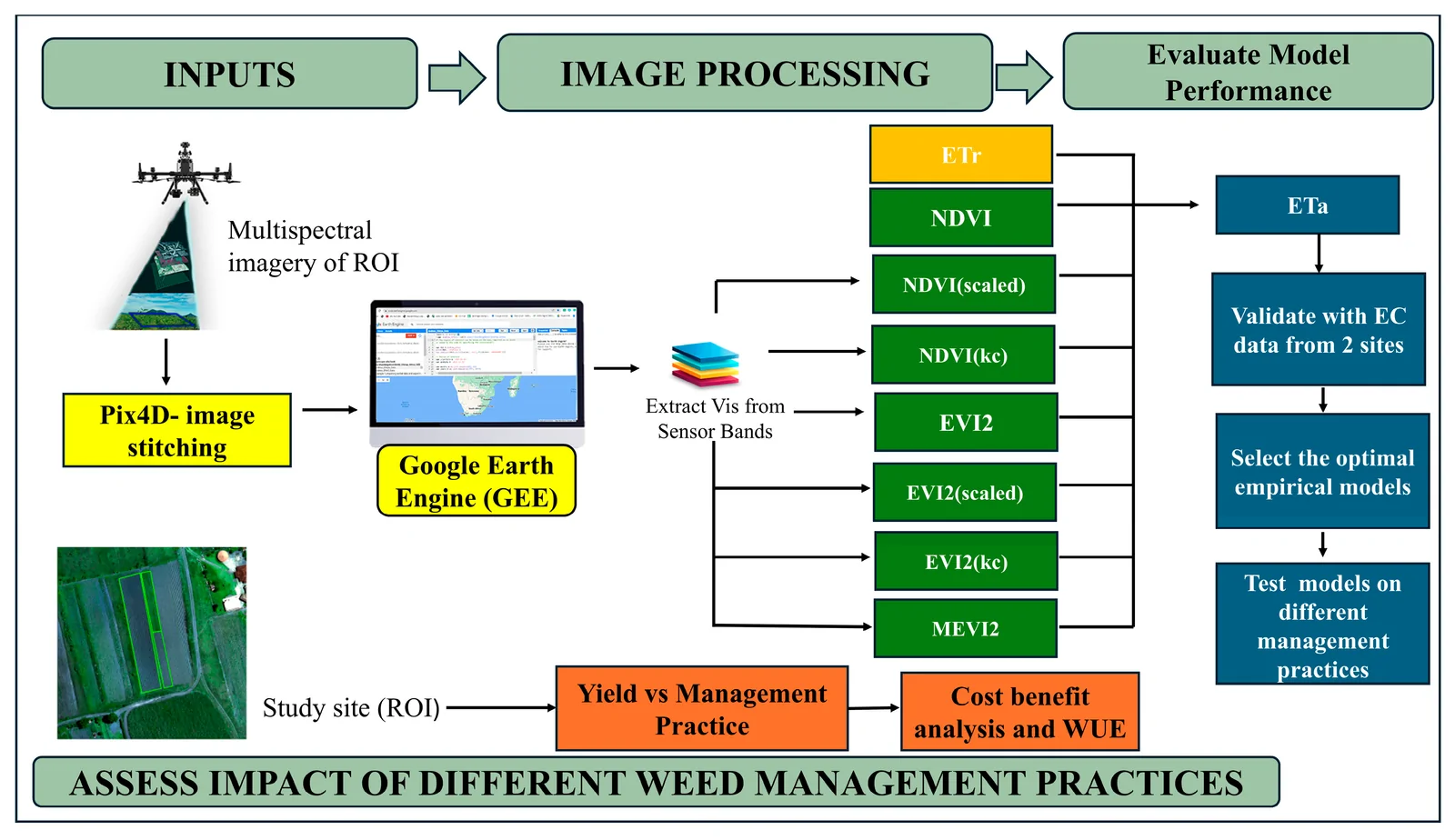
Flowchart outlines the methodological approach used in this study.

The costs and benefits of the different weed management practices were assessed. The average corm mass per plant was measured at the end of the growing season across different plots. This allowed the estimation of the total yield per plot, assuming the average corm mass per plant remained constant within a particular field. The plot area and number of plants were standardized. The intermediate field was chosen as the reference for this investigation, covering approximately 1300 m^2^. It consisted of 17 rows, each with about 196 plants, allowing for a total capacity of 3332 plants. The price of taro per kilogram was estimated using yield data from the intermediate field and information provided by the farmer. According to the farmer, a 20 kg batch of taro was sold for ZAR 130.00 (approximately USD 7.40), which is equivalent to ZAR 6.50 per kg, or approximately USD 0.37 per kg (based on current exchange rates). To assess the observed association of management practices and yield, the average mass of 5 samples from each of the three plots was determined. In calculating yield per unit area, it was assumed that all plants interact uniformly with the environment across the field. A summarized flowchart depicting the methodology that was adopted for this study is illustrated below in Figure 12.

## 5. Conclusions

The ETa-MEVI2 model using UAV imagery was identified as the optimal approach for estimating water use of rainfed taro. When applied across plots with different weed control practices, the results show that the weeded field used 27.0% less water, while the unweeded field used 35.5% more water than the farmer-managed (intermediate) plot. Although the unweeded plot represents a combined ETa of mixed vegetation and bare soil and not the crop alone, these findings highlight the model’s capability to observe associations between management practices and variation in ETa estimates, making it a potentially valuable tool for arid and semi-arid regions with limited resources. The high spatial resolution provided by multispectral UAV imagery enabled detailed ETa mapping, revealing intra-field variability. The framework underscores the model’s potential utility for site-specific decision-making in land and water resource management for extension officers and decision-makers. However, there is still a need to address the limitations of this study to confirm whether the observed differences can be attributed exclusively to weed management practices. It is also important to acknowledge the digital divide that exists between researchers and smallholder farmers, especially in the context of sub-Saharan Africa. Therefore, strategic collaboration between research institutions and government agencies is vital for establishing capacity-building infrastructure that bridges existing gaps and addresses practical challenges, such as the high cost of UAV technology and the specialized skills required to operate them effectively.

Furthermore, these tools can contribute to climate adaptation strategies by helping farmers optimize water use under changing rainfall and temperature patterns. Future research should explore integrating remote sensing-based ETa models with gridded and in situ meteorological data to enhance our understanding and their applicability in areas lacking weather stations. Refining the empirical relationships between vegetation indices and crop coefficient (Kc) across growth stages and diverse agroecological zones will improve scalability and seasonal adaptability.

## Figures and Tables

**Table 1:** The results of accuracy metrics for observed ETa data captured by the EC flux tower and the estimated ETa from the VI-based models.

Site	Statistic	ETa-NDVI	ETa-NDVI Scaled	ETa-NDVI (Kc)	ETa-EVI2	ETa-EVI2 Scaled	ETa-EVI2 (Kc)	ETa-MEVI2
Site A	R^2^	0.83	0.83	0.84	0.83	0.83	0.86	0.87
	MAE	0.38	0.47	1.49	1.03	0.70	0.56	0.63
	RMSE	0.44	0.56	1.66	1.14	0.78	0.60	0.68
	Score	0.800	0.728	0.050	0.336	0.573	0.833	0.831
	Rank	3	4	7	6	5	1	2
Site B	R^2^	0.49	0.49	0.49	0.49	0.51	0.57	0.57
	MAE	0.58	1.20	0.79	0.66	0.59	0.76	0.58
	RMSE	0.76	1.36	0.95	0.83	0.79	0.95	0.80
	Score	0.800	0.000	0.538	0.702	0.824	0.757	0.973
	Rank	3	7	6	5	2	4	1

**Table 2:** Site A (irrigated, cannabis): percentage of days within the ±20% tolerance band, cumulative bias, and cumulative ETo, and ETa relative to measured ETa, by growth phase and for the full season (S1 = day 42–72, S2 = day 73–114, S3 = day 115–160, full season = day 42–160).

Model	S1				S2				S3				Full Season		
	% Within ±20%	Cum. Bias (%)	ETa (mm)	ETo (mm)	% Within ±20%	Cum. Bias (%)	ETa (mm)	ETo (mm)	% Within ±20%	Cum. Bias (%)	ETa (mm)	ETo (mm)	% Within ±20%	Cum. Bias (%)	ETa (mm)
ETa-EC direct (measured)	-	-	101.3	134.5	-	-	127.8	167.2	-	-	107.0	162.5	-	-	336.2
ETa-NDVI	38.7	−30.5	70.5	134.5	33.3	−24.8	96.2	167.2	47.8	−12.8	93.3	162.5	40.3	−22.7	259.9
ETa-NDVI scaled	67.7	−16.6	84.6	134.5	78.6	−9.8	115.4	167.2	60.9	+4.7	112.0	162.5	68.9	−7.2	311.9
ETa-NDVI (Kc)	19.4	+13.5	115.0	134.5	40.5	+20.2	153.6	167.2	28.3	+39.4	149.2	162.5	30.3	+24.3	417.8
ETa-EVI2	0.0	−58.2	42.4	134.5	0.0	−46.7	68.1	167.2	37.0	−31.4	73.4	162.5	14.3	−45.3	183.8
ETa-EVI2 scaled	0.0	−49.8	50.8	134.5	0.0	−36.1	81.7	167.2	47.8	−17.7	88.0	162.5	18.5	−34.4	220.6
ETa-EVI2(Kc)	67.7	−21.2	79.9	134.5	81.0	−7.3	118.6	167.2	47.8	+16.1	124.2	162.5	64.7	−4.0	322.6
ETa-MEVI2	67.7	−20.5	80.6	134.5	88.1	−3.4	123.5	167.2	41.3	+20.6	129.1	162.5	64.7	−0.9	333.1

**Table 3:** Site B (rainfed, taro): percentage of days within the ±20% tolerance band, cumulative bias, cumulative ETo and estimated ETa relative to measured ETa, by growth phase and for the full season (Phase II = day 78–196, Phase III = day 197–221, full season = day 78–221).

Model	Phase II				Phase III				Full Season		
	% within ±20%	Cum. Bias (%)	ETa (mm)	ETo (mm)	% within ±20%	Cum. Bias (%)	ETa (mm)	ETo (mm)	% within ±20%	Cum. Bias (%)	ETa (mm)
ETa-EC direct (measured)	-	-	151.2	347.1	-	-	17.5	59.8	-	-	168.8
ETa-NDVI	50.4	+14.9	173.7	347.1	16.0	−10.7	15.7	59.8	44.4	+12.2	189.4
ETa-NDVI scaled	14.3	+37.8	208.5	347.1	28.0	+7.1	18.8	59.8	16.7	+34.7	227.3
ETa-NDVI (Kc)	5.0	+89.5	286.6	347.1	16.0	+79.8	31.5	59.8	6.9	+88.5	318.1
ETa-EVI2	7.6	−23.7	115.4	347.1	8.0	−49.8	8.8	59.	7.6	−26.4	124.2
ETa-EVI2 scaled	22.7	−9.5	136.9	347.1	8.0	−40.7	10.4	59.8	20.1	−12.7	147.3
ETa-EVI2 (Kc)	12.6	+41.3	213.7	347.1	48.0	+31.0	23.0	59.8	18.8	+40.2	236.6
ETa-MEVI2	16.8	+13.8	172.1	347.1	28.0	−14.4	15.0	59.8	18.8	+10.9	187.1

**Table 4:** The correlation between Kc and the VI-based proxies during different growth phases.

ROI	Growth Phase	DAP Range	Kc	MEVI2	NDVI	NDVI (Scaled)	EVI2 (Kc)	EVI2 (Scaled)
Site A	S1	42–72	0.75	0.60	-	0.63	0.59	-
	S2	73–114	0.76	0.74	-	0.69	0.71	-
	S3	115–160	0.66	0.79	-	0.69	0.76	-
Site B	Phase II	78–196	0.44	0.50	0.50	-	-	0.39
	Phase III	197–221	0.29	0.25	0.26	-	-	0.17

**Table 5:** The ETa estimates for plots with different weed management practices using the ETa-MEVI2 model on days when UAV imagery was captured.

				ETa-MEVI2					
Image	Day of Year	DAP	ETo (mm)	UNWEEDED		WEEDED		INTERMEDIATE	
				MEVI2	ETa (mm)	MEVI2	ETa (mm)	MEVI2	ETa (mm)
a	22 November 2023	62	6.90	0.67	4.65	0.12	0.80	0.12	0.79
b	8 December 2023	78	6.98	0.98	6.87	0.37	2.61	0.33	2.31
c	19 December 2023	89	1.59	1.03	1.64	0.58	0.93	0.49	0.78
d	10 January 2024	111	3.74	0.55	2.05	0.28	1.04	0.28	1.04
e	25 January 2024	126	2.08	1.01	2.10	0.75	1.55	0.87	1.81
f	6 February 2024	138	6.57	0.92	6.07	0.81	5.36	0.94	6.18
g	21 February 2024	153	5.63	0.92	5.18	0.62	3.50	1.01	5.68
h	15 March 2024	175	5.50	0.77	4.23	0.42	2.32	0.96	5.26
i	26 March 2024	186	5.44	1.01	5.51	0.67	3.65	0.34	1.83

**Table 6:** The distribution of simulated crop water use in plots where taro was grown under different weed management practices during different growth phases.

Model	Growth Phase	DAP Range	Unweeded (mm)	Weeded (mm)	Intermediate (mm)
ETa-MEVI2	Phase I	50–59	27.49	4.70	4.69
	Phase II	60–179	382.26	221.06	289.81
	Phase III	180–191	29.91	16.40	37.24

**Table 7:** The changes in MEVI2 as a proxy for Kc, during different growth phases, in plots where different weed management practices were implemented.

Kc proxy	Growth Phase	DAP Range	ETo (mm)	Unweeded Field	Weeded Field	Intermediate
**MEVI2**	Phase I	50–59	40.79	0.67	0.12	0.12
	Phase II	60–179	440.62	0.87	0.51	0.66
	Phase III	180–191	38.91	0.77	0.42	0.96

**Table 8:** Management expenses associated with the different practices during the growth of taro.

Weed Management Practice	Input	Cost (ZAR)	Cost (USD)
Unweeded	Cultivator	R 300	$16.67
	no extra input	R 0	$0
	Total	R 300	$16.67
Weeded	Cultivator	R 300	$16.67
	weeding (9 times)	R 5400	$300
	Total	R 5700	$316.67
Intermediate	Cultivator	R 300	$16.67
	Herbicide and application (R1782 + R 300)	R 2082	$115.67
	Cattle cultivation	R 480	$26.67
	Manual labor	R 600	$33.33
	Total	R 3462	$192.33

**Table 9:** Corm mass per plant across the different weed management practices based on the 5 samples per plot.

Management Practice	Sample 1 (g)	Sample 2 (g)	Sample 3 (g)	Sample 4 (g)	Sample 5 (g)	Mean ± SD (g)
Unweeded	143.7	168.4	151.6	174.3	143.0	156.2 ± 14.4
Weeded	982.4	1054.7	1011.3	1038.6	1001.0	1017.6 ± 29.0
Intermediate	756.6	821.3	783.7	806.4	780.0	789.6 ± 25.0

**Table 10:** The potential yield, net profit margins, and water use efficiency (WUE) from the different weed management practices.

Management Practice	Corm Mass Per Plant (g)	Number of Plants in a Field	Mass of Corms Per Field (kg)	Revenue Per Field	Management Expenses	Net Profit (ZAR)	Net Profit (USD)	WUE (kg/mm)
Unweeded	156.2	3332	520.46	R 3383	R 300	R 3083	$171	1.16
Weeded	1017.6	3332	3390.64	R 22039	R 5700	R 16339	$908	14.00
Intermediate	789.6	3332	2630.95	R 17101	R 3462	R 13639	$758	7.93

**Table 11:** The cumulative rainfall, average relative humidity (RH avg), and minimum and maximum air temperatures (TMIN and TMAX obtained from eddy covariance (EC) systems located near the three study sites during the respective monitoring periods: Site A (14 December 2022 to 12 April 2023), Site B (1 January 2022 to 11 April 2022), and site C (22 November 2023 to 31 March 2024)).

ROI	Measurement Period (Days)	TMAX Avg (°C)	TMIN Avg (°C)	Rainfall (mm)	RH Avg (%)
Site A	120	25.55	15.86	294.60	73.68
Site B	101	26.57	16.31	292.60	67.41
Site C	131	24.93	16.60	250.50	82.35

**Table 12:** The crop development stages of cannabis according to Denton et al. [57] and Mediavilla et al. [58].

Growth Phase	Development	Duration (Weeks After Planting)
S0	Germination and emergence	≈1–4 weeks
S1	Vegetative stage	≈5–9 weeks
S2	Full canopy flowering/bud formation, peak water demand	≈10–14weeks
S3	Flower maturation, leaf senescence	≈15–20 weeks

**Table 13:** The crop development stages of taro. This information was supported by other studies [1,59,60].

Growth Phase	Development Stage	Months After Planting
Phase I (crop establishment)	Recovered plant after sowing	1–2 months
Phase II (grand growth)	Maximum rooting depth, and canopy cover	4- 5 months
Phase III (Maturation)	Leaf senescence, yield formation	6–8 months

**Table 14:** The sensors mounted on the eddy covariance flux tower in sites A and B.

Instruments	Measurement	Manufacturer
EC150 CO_2_/H_2_O Open-Path Gas Analyser	CO_2_/H_2_O flux	Campbell Scientific, Logan, UT, USA
TE525 mm Tipping Bucket Rain Gauge	Rainfall	Texas Instruments, Dallas, TX, USA
HC2S3 Temperature and Relative Humidity Probe	Temperature; relative humidity	Campbell Scientific, Logan, UT, USA
HFP01 Soil Heat Flux Plate	Ground heat flux	Huxaflux, Delft, The Netherlands
CSAT3A Three-Dimensional Sonic Anemometer	CO_2_/H_2_O flux	Campbell Scientific, Logan, UT, USA
CNR4 Net Radiometer	Net solar radiation	Kipp and Zonen, Delft, The Netherlands
FW1 Type E fine wire thermocouples	Air temperature	Campbell Scientific, Logan, UT, USA
TCAV Type E thermocouples	Average soil temperature	Campbell Scientific, Logan, UT, USA
CS616 Water Content Reflectometers	Volumetric soil water content	Campbell Scientific, Logan, UT, USA

**Table 15:** Specifications of the sensors that captured the imagery used in this study.

Sensor	Band	Central Wavelength (nm)	GSD (m.pixel^−1^)
Mica Sense Altum Sensor	B1—Blue	475	0.07
	B2—Green	560	0.07
	B3—Red	668	0.07
	B4—Red-edge	717	0.07
	B5—Near-infrared (NIR)	842	0.07
	B6—Thermal infrared	8000–14,000	1.09
Mavic 3 multispectral	B2—Green	560	0.007
	B3—Red	650	0.007
	B4—Red-edge	730	0.007
	B5—Near-infrared (NIR)	860	0.007

## Data Availability

The data presented in this study are available upon request from the corresponding author.
